# Botanical and Upcycled Bioactives for Advanced Topical Formulations: Mechanistic Pathways, Cutaneous Delivery, and Sustainability-by-Design

**DOI:** 10.3390/pharmaceutics18030375

**Published:** 2026-03-18

**Authors:** Salvatore Panza, Beatrice Pellegrini, Dorotea Fiore, Martine Tarsitano, Antonia Mancuso, Maria Chiara Cristiano, Donatella Paolino

**Affiliations:** 1 Department of Experimental and Clinical Medicine, University of Catanzaro “Magna Graecia”, Viale “S. Venuta s.n.c.”, I-88100 Catanzaro, Italy; beatrice.pellegrini@studenti.unicz.it (B.P.); martine.tarsitano@studenti.unicz.it (M.T.); antonia.mancuso@unicz.it (A.M.); paolino@unicz.it (D.P.); 2 Research Center “ProHealth Translational Hub”, University of Catanzaro “Magna Graecia”, Viale “S. Venuta s.n.c.”, I-88100 Catanzaro, Italy; 3 Department of Medical and Surgical Sciences, University of Catanzaro “Magna Graecia”, Viale “S. Venuta s.n.c.”, I-88100 Catanzaro, Italy; dorotea.fiore@studenti.unicz.it (D.F.); mchiara.cristiano@unicz.it (M.C.C.)

**Keywords:** botanical ingredients, natural cosmetics, advanced delivery systems, skincare formulations, sustainable dermatology, regulatory frameworks

## Abstract

Natural and sustainable cosmetics represent a rapidly evolving frontier in dermatological science, integrating plant-derived bioactive compounds with advanced delivery technologies and environmentally conscious formulation design. Botanical ingredients, including polyphenols, flavonoids, terpenoids, alkaloids, and polysaccharides, modulate key biological pathways involved in oxidative stress, inflammation, extracellular matrix remodeling, pigmentation, and immune responses, thereby supporting skin regeneration, protection, and homeostasis. To overcome limitations related to instability, compositional variability, and limited skin penetration, these compounds are increasingly incorporated into advanced delivery systems such as nanoemulsions, solid lipid nanoparticles (SLNs), nanostructured lipid carriers (NLCs), vesicular systems, microneedle platforms, three-dimensional matrices, and plant-derived extracellular vesicles (PDEVs). These technologies enhance cutaneous bioavailability, enable controlled release, and improve tissue targeting, linking formulation design to exposure–response relationships. In parallel, sustainability has become a critical component of product development. Circular economy strategies, including the upcycling of agro-industrial by-products, green extraction technologies, biodegradable packaging, and life cycle assessment, are reshaping cosmetic innovation. Regulatory frameworks are also evolving to address safety, efficacy, and transparency of natural claims, as well as the challenges of botanical standardization. This narrative review, conducted through a structured literature search, provides a mechanistically oriented analysis of botanical ingredients in dermatology, emphasizing molecular pathways, skin delivery science, and safety considerations. Rather than cataloguing ingredients, it proposes a translational framework linking phytochemistry, delivery science, safety-by-design principles, and sustainability to support the rational development of effective and safe dermatological formulations.

## 1. Introduction

Natural resources have historically been employed in skincare for their healing, soothing, and protective effects. Ancient medical traditions such as Traditional Chinese Medicine and Ayurveda used herbal extracts, essential oils, and mineral clays for dermatological purposes [1,2]. Contemporary science has validated this ethnobotanical knowledge through phytochemical and pharmacological studies, confirming the biological activity of numerous plant-derived compounds. Among these, tannins, alkaloids, peptides, and flavonoids are among the most promising candidates due to their antioxidant, anti-inflammatory, antimicrobial, photoprotective, and regenerative properties [3,4].

In parallel, the cosmetic industry has undergone a profound transformation, driven by increasing consumer demand for safer, multifunctional, and environmentally responsible products [5,6]. Natural and sustainable cosmetics, often formulated with plant-derived bioactive compounds, are now positioned as effective and sustainable alternatives to conventional formulations, combining perceived safety, biological efficacy, and consumer trust [7,8,9]. Market analyses indicate a steady growth of the global cosmetics sector, with increasing expansion of the natural and organic segment, highlighting the growing relevance of plant-based and sustainability-oriented products [7,10].

The emergence of cosmeceuticals, hybrid products positioned between cosmetics and pharmaceuticals, has further reshaped the skincare landscape. First introduced by Raymond Reed and later conceptually expanded by Dr. Albert Kligman in the 1970s, cosmeceuticals are designed to exert biological effects beyond simple aesthetic improvement, targeting cellular and molecular pathways involved in skin homeostasis, to reduce inflammation, delay aging, and promote tissue repair [11,12]. In this context, botanical cosmeceuticals have demonstrated the ability to modulate key molecular mechanisms, including collagen synthesis, inhibition of matrix metalloproteinases (MMPs), regulation of melanogenesis, attenuation of oxidative stress, and modulation of inflammatory signaling pathways [13,14,15,16]. However, despite extensive documentation of these effects, a critical and integrated evaluation of their mechanistic relevance in dermatology remains limited. A major challenge in the clinical and cosmetic translation of natural bioactives is their limited bioavailability, which often arises from unfavorable physicochemical properties such as poor water solubility, instability to light or heat, and restricted skin penetration. For instance, vitamin C (ascorbic acid), although a potent antioxidant, is highly water-soluble and chemically unstable, resulting in limited cutaneous retention and rapid degradation [17,18]. Conversely, lipid-soluble compounds such as vitamin E (tocopherol) readily integrate into cell membranes but may exhibit suboptimal penetration into deeper skin layers [19]. Moreover, specific skin cell populations, including melanocytes, are particularly susceptible to oxidative damage due to their intrinsically lower levels of endogenous antioxidant enzymes, further emphasizing the need for optimized delivery strategies [20,21].

To address these limitations, advanced skin delivery systems have been developed to enhance solubility, stability, penetration, and controlled release of botanical bioactives. Lipid-based nanocarriers, including SLNs, NLCs, nanoemulsions, as well as vesicular systems such as liposomes, niosomes, ethosomes and transferosomes have shown significant advantages in topical and transdermal formulations [22,23,24,25,26]. Beyond improving physicochemical performance, these systems actively influence bioactive-skin interactions by modulating cellular uptake mechanisms, local bioavailability, and interactions with inflammatory and oxidative signaling pathways. More recently, PDEVs have emerged as innovative biocompatible, and biodegradable delivery platforms capable of transferring proteins, RNA species, and secondary metabolites into skin cells, thereby contributing to immune modulation, redox balance, and tissue regeneration processes [27,28,29].

Alongside technological advances, sustainability has become a central driver in dermatological and cosmetic research, reflecting both consumer expectations and regulatory pressures. Circular economy models, particularly the upcycling of agro-industrial by-products into high-value cosmetic ingredients, are increasingly supported by green extraction technologies, biodegradable materials, and life-cycle assessments (LCA) approaches aimed at minimizing environmental impact [30,31,32]. However, botanical extracts remain chemically complex and subject to significant variability linked to geographical origin, cultivation practices, harvesting, and extraction methods. The standardization of phytochemical profiles and bioactive content is therefore essential to ensure reproducibility and safety [33,34]. At the same time, concerns about greenwashing-the deceptive marketing of products as “natural” or “eco-friendly” without robust evidence-have prompted international organizations to introduce certification frameworks that strengthen transparency and consumer trust [35,36,37].

This review provides an integrated and structured narrative approach to critically examine the role of botanical and upcycled bioactives in modern dermatology. It focuses on molecular mechanisms of action, advanced skin delivery strategies, dermatological applications, sustainability principles, and regulatory considerations. Rather than cataloguing botanical ingredients or their biological activities in isolation, this review explicitly connects pharmacological mechanisms with formulation science, sustainability-driven valorization, and regulatory constraints. By adopting this integrative perspective, the present work moves beyond descriptive overviews and proposes a translational framework that links phytochemistry, delivery science, safety-by-design, and sustainability to support the development of next-generation natural and sustainable dermatological and cosmetic products.

## 2. Methods

This narrative and integrative review was conducted using a structured and transparent literature search strategy aimed at critically analyzing botanical and upcycled bioactive ingredients for dermatological and cosmetic applications, with particular emphasis on molecular mechanisms, skin delivery strategies, sustainability, and regulatory aspects.

A comprehensive search was performed across multiple scientific databases, including PubMed, Scopus, Web of Science, and Google Scholar. Peer-reviewed articles published predominantly within the last 10 years were considered, with priority given to studies from 2020 onward, in order to capture recent advances in skin biology, formulation science, and sustainability-driven cosmetic innovation.

Search queries were developed using combinations of controlled vocabulary and free-text terms related to the main thematic pillars of the review, including:

- Botanical and plant-derived bioactives (e.g., polyphenols, flavonoids, carotenoids, essential oils);
- Dermatological applications and skin biology (e.g., skin barrier, inflammation, photoaging, pigmentation disorders, wound healing);
- Topical and advanced skin delivery strategies (e.g., nanoemulsions, lipid-based carriers, nanostructured lipid carriers, solid lipid nanoparticles, extracellular vesicles);
- Sustainability-oriented concepts (e.g., upcycling, agro-industrial by-products, circular economy, green extraction technologies).

Boolean operators (AND/OR) were applied to refine the search and ensure relevance.

Original studies were prioritized when discussing mechanistic insights, formulation performance, skin penetration, and biological efficacy, while review articles were used to contextualize the state of the art, consolidate established mechanisms, and identify emerging trends within the field. The literature search primarily focused on peer-reviewed scientific publications addressing mechanistic, pharmacological, and formulation aspects of botanical bioactives in dermatology. Patent databases were not systematically included, as the objective of the review was to analyze experimental and translational evidence reported in the scientific literature.

To address safety assessment and regulatory considerations, targeted searches were conducted on international cosmetic regulations and guidance documents, including the European Union Cosmetic Regulation (EC No. 1223/2009), relevant OECD Test Guidelines and Integrated Approaches to Testing and Assessment (IATA), as well as regulatory frameworks from other major markets such as the United States (FDA), China (NMPA), and Japan (MHLW). These sources were used to contextualize toxicological evaluation strategies, skin sensitization assessment, and global compliance requirements for botanical and upcycled cosmetic ingredients.

Articles were excluded if they were unrelated to topical dermatological or cosmetic applications, lacked sufficient methodological detail, or were not written in English. Following title and abstract screening, full-text articles were evaluated based on scientific quality, relevance to the review objectives, and consistency with regulatory and safety considerations. This approach ensured thematic coherence while allowing integration across pharmacology, formulation science, sustainability, and regulatory frameworks.

## 3. Botanical Bioactives in Dermatology: Mechanisms, Translational Barriers and Delivery Strategies

Botanical ingredients, traditionally employed in folk medicine, have increasingly become the focus of dermatological research due to their broad spectrum of bioactivities and generally favorable safety profiles when properly standardized. Plant-derived secondary metabolites, including polyphenols, carotenoids, terpenoids, alkaloids, polysaccharides, and lipids exert antioxidant, anti-inflammatory, antimicrobial, wound-healing, photoprotective, and anti-aging effects through the modulation of multiple biological targets in the skin [15,38,39,40]. Their relevance in dermatology lies in their ability to counteract oxidative stress, modulate inflammatory pathways, regulate melanogenesis, preserve extracellular matrix (ECM) homeostasis, and reinforce the skin barrier function. Beyond their biological activity, botanical ingredients may also contribute to formulation performance by enhancing stability and supporting multifunctional product design, while aligning with the growing demand for sustainable and naturally derived cosmetic and dermatological products [41]. However, contemporary evidence highlights that their effective translation into clinically relevant outcomes critically depends on standardized extraction procedures, rigorous quality control, and the implementation of advanced delivery platforms capable of improving bioavailability and ensuring reproducible biological response [4].

### 3.1. Integrated Molecular Signaling Networks Underlying Botanical Bioactivity

The biological activity of botanical compounds in dermatology cannot be fully interpreted by considering individual pathways in isolation, as redox, inflammatory, and stress-response networks are tightly interconnected. Reactive oxygen species (ROS) act as central signaling mediators, activating mitogen-activated protein kinase (MAPK) cascades, including ERK, JNK and p38, which regulate transcription factors such as AP-1 and NF-κB. This signaling axis promotes the expression of matrix metalloproteinases (MMPs), pro-inflammatory cytokines, and enzymes such as COX-2 and iNOS, contributing to extracellular matrix degradation and chronic inflammation [20,42,43,44,45].

In parallel, oxidative stress induces activation of the nuclear factor erythroid 2-related factor 2/antioxidant response element (Nrf2/ARE) pathway, leading to upregulation of cytoprotective and detoxifying enzymes, including heme oxygenase-1 (HO-1), NAD(P)H quinone dehydrogenase 1 (NQO1), and other redox-regulating systems that limit oxidative damage and support cell survival [46,47,48,49,50]. Importantly, Nrf2 signaling exhibits functional antagonism toward NF-κB-mediated inflammation, illustrating a redox-dependent balance between cytoprotection and inflammatory activation [45,47]. Botanical polyphenols and carotenoids frequently modulate this equilibrium by attenuating ROS levels while directly influencing kinase activity and transcriptional responses [44,51,52,53,54,55].

Additional regulatory layers involve the SIRT1 and PI3K/Akt pathways, which connect mitochondrial function, cellular energy homeostasis, and survival signaling. Activation of these axes has been associated with enhanced DNA repair, improved mitochondrial resilience, and delayed cellular senescence in UV-stressed skin cells [56,57,58]. The convergence of these pathways explains the pleiotropic dermatological effects of botanical bioactives, including antioxidant protection, anti-inflammatory activity, photoprotection, preservation of extracellular matrix integrity, and support of tissue regeneration [55,59,60,61].

**Figure 1:**
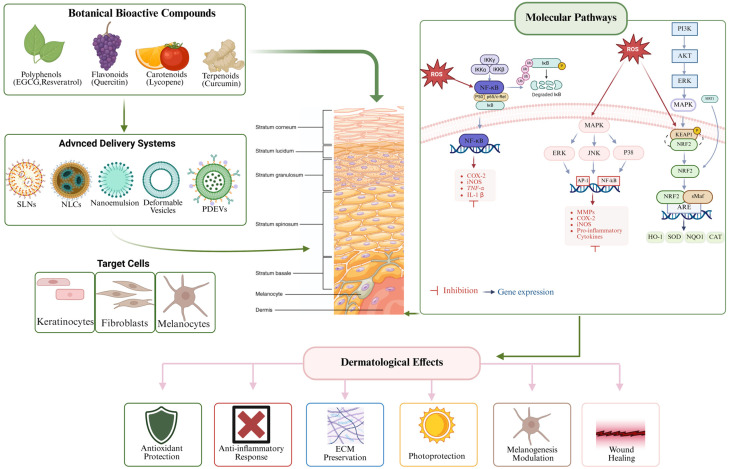
Mechanistic pathways and advanced delivery strategies of botanical bioactive compounds in dermatological applications. The schematic illustration summarizes the integration of botanical bioactives, advanced topical delivery systems, and their molecular effects in skin cells. Representative plant-derived compounds, including polyphenols (e.g., epigallocatechin gallate and resveratrol), flavonoids (e.g., quercetin), carotenoids (e.g., lycopene), and terpenoids (e.g., curcumin), can be incorporated into advanced delivery platforms such as SLNs, NLCs, nanoemulsions, deformable vesicles, and PDEVs. These systems facilitate enhanced penetration across the SC and targeted interaction with skin cells including keratinocytes, fibroblasts, and melanocytes. At the molecular level, botanical bioactives modulate key intracellular signaling pathways associated with oxidative stress and inflammation, including NF-κB, MAPK (ERK, JNK, p38), PI3K/AKT, and the Nrf2/Keap1 antioxidant response pathway. Through the regulation of these signaling networks, botanical compounds influence gene expression involved in inflammatory mediators, antioxidant defenses, and extracellular matrix remodeling. The resulting dermatological effects include antioxidant protection, anti-inflammatory activity, preservation of extracellular matrix integrity, photoprotection, modulation of melanogenesis, and promotion of wound healing. Created in BioRender. Cristiano, M. C. (2026). https://BioRender.com/7rjirmp, accessed on 11 March 2026.

Understanding this network-level cross-talk is therefore essential for interpreting the biological efficacy of plant-derived compounds and for designing delivery systems capable of achieving pharmacologically meaningful concentrations in target skin compartments in Figure 1.

#### 3.1.1. Antioxidant Activity and Its Relevance in Skin Aging

Skin aging is a complex and multifactorial process influenced by the interplay between intrinsic biological factors and extrinsic environmental stressors, with photoaging representing the most significant external driver. UV radiation is the primary contributor, High-Energy Visible (HEV) light, infrared radiation, and air pollution are increasingly recognized as aggravating factors [21,62]. These stressors promote ROS generation, mitochondrial dysfunction, and activation of transcription factors, including NF-κB and AP-1. The downstream consequences include the release of pro-inflammatory cytokines and upregulation of MMPs, which accelerate collagen degradation, extracellular matrix remodeling and wrinkle formation [42,43].

Botanical antioxidants counteract these processes through complementary and interconnected mechanisms. Several plant-derived compounds, including flavonoids, carotenoids, and phenolic acids act as direct ROS scavengers, thereby limiting oxidative damage at the cellular level [51,63,64]. Others chelate transition metals, thereby reducing Fenton-driven oxidative reactions [20,63,65]. In addition to these phytochemicals actions, carotenoids such as β-carotene and lycopene absorb UV light or quench singlet oxygen, extending photoprotection beyond that provided by conventional sunscreens [54,66].

Importantly, multiple studies demonstrate that botanical antioxidants modulate endogenous defense systems through activation of nuclear factor erythroid 2-related factor 2/antioxidant response element (Nrf2/ARE) pathway in skin cells [46,47]. In UVB-exposed human keratinocytes, plant-derived extracts and isolated phytochemicals have been shown to induce Nrf2 nuclear translocation and enhance the expression of cytoprotective enzyme, such as heme oxygenase-1 (HO-1), NAD(P)H quinone dehydrogenase 1 (NQO1), resulting in reduced oxidative DNA damage and improved cell viability [48,49,50]. These Nrf2-dependent responses concurrently attenuate ROS-driven activation of MAPK signaling cascades, thereby indirectly suppressing AP-1-mediated transcription of MMPs implicated in photoaging [44].

In parallel, inhibition of NF-κB signaling by botanical antioxidants has been experimentally linked to reduced production of pro-inflammatory cytokines and decreased MMP expression in UV-irradiated skin models, establishing a mechanistic connection between antioxidant activity, inflammation control, and preservation of dermal matrix integrity [45]. This coordinated regulation of oxidative and inflammatory pathways represents a key molecular basis for the anti-aging efficacy of botanical ingredients.

Lycopene, β-carotene, lutein, and astaxanthin consistently demonstrate protection effects against UV- and HEV-induced oxidative stress, with clinical and ex vivo studies reporting increased erythema thresholds and reductions in oxidative stress biomarkers following supplementation or topical application [54,55,59,67]. In particular, astaxanthin exhibits strong singlet oxygen-quenching capacity and has been shown in human epidermal keratinocytes to significantly reduce UVB-induced ROS production and apoptosis, providing functional evidence of its antioxidant mechanism [60,68]. However, its poor aqueous solubility and chemical instability have driven the development of advanced formulation strategies to enhance topical bioavailability. Beyond photoaging, botanical antioxidants have demonstrated efficacy against emerging environmental stressors.

Anti-pollution studies reveal that curcumin and piperine, when delivered via ethosomal systems, effectively protect human skin explants from diesel exhaust-induced oxidative damage, preserving cellular morphology and redox balance [69]. Together, these findings provide mechanistic and functional evidence supporting the central role of botanical antioxidants in mitigating photoaging and enhancing skin resilience against increasingly complex exposome conditions.

#### 3.1.2. Polyphenols in Dermatology

Polyphenols represent the most extensively studied class of botanical metabolites in dermatology, comprising flavonoids, stilbenes, phenolic acids, and lignans. Their dermatological relevance extends beyond generic antioxidant activity and is rooted in their ability to modulate intracellular signaling pathways in keratinocytes and dermal fibroblasts, thereby influencing inflammation, pigmentation, ECM remodeling, and cellular stress responses [44,52,53]. Experimental evidence indicates that polyphenols act as pleiotropic regulators, capable of reprogramming redox-sensitive and stress-activated pathways rather than functioning as simple radical scavengers [64,70]. These molecular actions reduce pro-inflammatory cytokines: interleukin-1 beta (IL-1β), interleukin-6 (IL-6), tumor necrosis factor alpha (TNF-α) and MMPs, thereby preserving ECM integrity and counteracting photoaging [61].

Among well-characterized polyphenols, epigallocatechin gallate (EGCG) from *Camellia sinensis* has been shown in UV-irradiated skin models to attenuate photoaging-related damage by suppressing matrix MMP expression and preserving collagen integrity. These effects have been mechanistically linked to the modulation of MAPK and AP-1 dependent signaling cascades, providing a molecular basis for the photoprotective and anti-aging properties of EGCG [61]. Importantly, such activities are observed in keratinocytes and dermal fibroblasts under oxidative stress conditions, supporting their relevance for cutaneous aging processes.

Resveratrol, a stilbene abundant in grapes and berries, exerts complementary dermatological effects through the activation of SIRT1-dependent pathways and the concomitant suppression of inflammatory signaling mediated by MAPK and NF-κB [56]. In skin-relevant cellular models, resveratrol has been reported to enhance cellular resistance to oxidative stress, improve mitochondrial function, and attenuate the inflammatory response associated with photoaging [57]. However, its clinical translation has been limited by poor stability and low skin penetration, prompting the development of nanocarrier-based formulations that significantly improve topical bioavailability and biological efficacy [71,72,73].

Flavonoids, constitute the largest and most structurally diverse subclass of polyphenols and are widely distributed in fruits, vegetables, tea, and medicinal plants [74]. Based on their C6-C3-C6 backbone and substitution patterns are classified into six groups: flavonols, flavones, flavanones, flavanols (catechins), isoflavones, and anthocyanins [75,76,77]. Structural features such as hydroxylation, methylation, and glycosylation significantly influence their redox behavior, stability, skin permeability, and affinity for molecular targets, thereby accounting for the heterogeneity of their dermatological effects [78]. Functionally, flavonoids modulate multiple pathways relevant to skin homeostasis. In addition to enhancing endogenous antioxidant defenses, selected flavonoids directly regulate pigmentation processes by down-regulating microphthalmia-associated transcription factor (MITF) and tyrosinase expression, providing a mechanistic rationale for their use in hyperpigmentation disorders and photo-induced dyschromia [79].

Phenolic acids, such as gallic and ferulic acid, provide complementary antioxidant and stabilizing effects, extending shelf life and bioactivity in cosmetic formulations [80]. Delivery technologies, including polymeric nanoparticles, SLNs, and NLCs, have successfully enhanced the solubility, stability, skin penetration and antioxidant activity of polyphenols [22,23,24]. Other flavonoids such as quercetin, kaempferol and apigenin exhibit combined antimicrobial and anti-inflammatory effects, further broadening their applicability in dermatological and wound-healing contexts [81,82].

Beyond redox and inflammatory modulation, polyphenols influence survival and repair-associated signaling pathways, PI3K/Akt and SIRT1. Activation of these pathways has been associated with enhanced DNA repair capacity, improved mitochondrial integrity, and delayed cellular senescence in UV-stressed skin cells [58,83]. The convergence of these molecular mechanisms highlights the multi-targeted nature of polyphenols, distinguishing them from single-action antioxidants and underscoring their relevance for advanced dermatological and cosmetic formulations.

### 3.2. Translational Challenges: Bioavailability, Variability, and Safety Barriers

Despite their considerable therapeutic and cosmetic potential, botanical ingredients face several critical challenges that limit their reproducibility, safety, and translational applicability. Key limitations include poor bioavailability, chemical instability, and insufficient skin penetration, particularly for polyphenols and carotenoids characterized by low aqueous solubility and sensitivity to light and heat [84]. In addition, variability in phytochemical composition arising from differences in plant species, geographical origin, cultivation practices, seasonal factors, and extraction methods represents a major obstacle to batch-to-batch consistency and reliable biological outcomes, as extensively discussed in safety-oriented reviews of botanical ingredients [33,34].

Beyond formulation-related challenges, safety and toxicological considerations remain a central and often underestimated issue in the use of botanical ingredients. Although often perceived as inherently safe, plant-derived compounds may cause adverse reactions, including skin sensitization, phototoxicity, and photoallergic responses, particularly when used at high concentrations or under UV exposure. These risks have been documented for several botanical extracts and natural cosmetic ingredients [85,86,87]. Moreover, the presence of contaminants, including pesticide residues, heavy metals, mycotoxins, and environmental pollutants, poses significant risks to product quality and consumer safety, highlighting the importance of rigorous raw material selection and analytical quality control, as emphasized by regulatory bodies and environmental monitoring studies [88,89,90].

To address these limitations, the standardization of active constituents and the implementation of advanced delivery strategies, such as lipid-based nanocarriers and extracellular vesicle-based systems, are increasingly recognized as essential tools for enhancing bioavailability while enabling controlled and safer exposure of skin tissues to bioactive compounds. However, the adoption of such technologies must be accompanied by robust safety assessment frameworks. Integrated Approaches to Testing and Assessment (IATA), together with in vitro and in silico methods for skin sensitization and phototoxicity evaluation, are now widely recommended to support risk assessment while reducing reliance on animal testing [91,92,93,94].

Looking forward, future research should prioritize the integration of green extraction technologies, upcycling strategies for agro-industrial by-products, and systems biology approaches, including transcriptomics, proteomics, and metabolomics, to elucidate molecular mechanisms of action while simultaneously addressing safety and efficacy [95,96,97,98]. In parallel, the harmonization of regulatory perspectives across major markets, including Europe, the United States, and Asia, has been identified as a key requirement for the global translation of botanical-based dermatological products [93,99,100]. Collectively, these advances will support the development of effective, safe, and genuinely sustainable botanical formulations, bridging scientific innovation, consumer expectations, and regulatory responsibility.

### 3.3. Advanced Skin Delivery Strategies for Botanical Ingredients

The following sections focus specifically on delivery systems as pharmacologically enabling platforms, addressing the translational limitations discussed above.

#### 3.3.1. Pharmacological and Biophysical Rationale for Advanced Skin Delivery

The dermatological translation of botanical bioactives is fundamentally constrained by the stratum corneum (SC), whose highly ordered, lipid-rich lamellar domains impose strict physicochemical requirements for penetration and intradermal distribution [101,102]. Many phytochemicals with strong dermatological potential, particularly polyphenols, flavonoids, and carotenoids, exhibit suboptimal permeation determinants (e.g., high polarity, unfavourable partitioning, limited aqueous solubility) and are frequently vulnerable to photo-oxidation and thermal degradation, leading to inconsistent exposure at viable epidermal and superficial dermal targets [84]. From a pharmacological standpoint, this translates into a failure to achieve the local concentration-time profiles required to modulate redox robustly and inflammation-dependent networks (e.g., Nrf2/ARE, NF-κB, MAPKs). Topical efficacy is therefore critically dependent on achieving sufficient local exposure at the site of action within viable skin [22,103].

Accordingly, advanced delivery systems should be framed as pharmacologically enabling platforms rather than formulation accessories, because they can actively reshape the drug-skin interaction by modulating (i) thermodynamic activity of the payload, (ii) SC hydration and lipid packing, (iii) appendageal deposition (follicular targeting), and (iv) intra-cutaneous residence time [101]. In practice, these effects are typically demonstrated in Franz diffusion cell experiments, tape-stripping/skin layer quantification, and microscopy-based bio-distribution (e.g., confocal imaging), which collectively quantify not only permeation flux but also skin retention and compartmentalization, a key determinant for topical efficacy and safety [104,105,106].

Importantly, several studies illustrate that delivery platforms can convert chemically fragile botanical fractions into reproducible dermal actives by improving stability and cutaneous exposure [25,26,107,108]. For example, SLNs carrying polyphenol-rich fractions from upcycled vine canes were shown to support anti-aging cellular outcomes by coupling stabilization and effective cell exposure, enabling measurable protection in skin-relevant in vitro models [109]. Similarly, nano-delivery approaches have been utilized to enhance epidermal bioavailability of canonical botanical antioxidants such as EGCG, where improved cutaneous performance is linked to carrier-controlled delivery and protection from oxidative stress [110]. These examples align with broader evidence that nanostructured systems can reduce formulation-driven variability and strengthen the exposure-response relationship for topical botanicals. Finally, delivery design also influences risk management. By promoting skin-restricted distribution (epidermal/upper dermal localization) and limiting uncontrolled surface accumulation or deeper systemic passage, advanced carriers can support a safety-by-design rationale, particularly relevant when botanical extracts contain multiple constituents with heterogeneous permeability and reactivity. In this context, advanced delivery systems help address the translational gap between robust in vitro bioactivity and variable clinical outcomes by enabling predictable, compartment-specific exposure in human skin models [22,103]. However, despite encouraging findings, many clinical studies on botanical dermatological formulations remain limited by small sample sizes, short treatment durations, formulation heterogeneity, and a lack of standardized outcome measures, which complicates direct comparison and generalization of results.

#### 3.3.2. Lipid-Based Nanocarriers: Mechanistic Determinants of Skin Penetration and Dermal Bioavailability

Lipid-based nanocarriers, including SLNs, NLCs, and nanoemulsions, have emerged as key enabling platforms for the dermal delivery and local pharmacokinetic control of botanical compounds whose intrinsic physicochemical properties limit passive skin permeation [111]. Their efficacy is not merely attributable to enhanced diffusion, but rather to a combination of barrier modulation, controlled release, and skin compartment targeting [22,107].

Recent ex vivo studies employing human skin Franz diffusion cells coupled with tape stripping and confocal microscopy [112] have demonstrated that lipid nanoparticles induce a pronounced occlusive and hydration-mediated effect, increasing stratum corneum hydration and transiently disrupting lipid lamellae, thereby facilitating intercellular penetration without compromising barrier integrity [22,106]. This mechanism has been shown to selectively enhance the accumulation of polyphenols in the epidermis and superficial dermis while minimizing systemic exposure [22,103].

Among lipid carriers, NLCs consistently outperform SLNs due to their less ordered lipid matrix, which improves loading efficiency and prevents drug expulsion during storage [107]. Quercetin-loaded NLCs have been experimentally shown to enhance topical performance, including increased deposition within specific skin compartments in in vitro/ex vivo models, supporting higher local bioavailability compared with conventional (non-nanostructured) formulations. Moreover, NLCs may offer formulation advantages over SLNs for quercetin, as reported by higher entrapment efficiency in direct NLC-SLN comparisons [113,114,115]. Similarly, resveratrol-loaded nanostructured lipid carrier hydrogels have been shown to improve the physicochemical stability and cutaneous accumulation of resveratrol, leading to enhanced protection against UV-induced oxidative stress and free radical formation compared with non-encapsulated formulations. For instance, a nanostructured lipid carrier gel loaded with resveratrol exhibited increased epidermal deposition and antioxidant efficacy in skin models under UV exposure [71,116]. These findings indicate that lipid nanocarriers enhance delivery efficiency, improve the effective cutaneous bioavailability of phytochemicals, and support pharmacodynamically relevant activity at target skin sites.

#### 3.3.3. Vesicular Systems: Deformability-Driven Transport and Epidermal Targeting

Vesicular nanocarriers represent a mechanistically distinct class of dermal delivery systems in which membrane fluidization and vesicle deformability are exploited to overcome the diffusional constraints imposed by the stratum corneum [117,118]. Unlike conventional liposomes, which mainly act as superficial reservoirs, ultradeformable vesicles such as ethosomes, transferosomes and related systems are engineered to modulate SC lipid organization and enable penetration into viable epidermal layers while maintaining barrier integrity [117,118,119].

Ethosomes, characterized by a high ethanol content, induce reversible fluidization of SC lipid and increased vesicle flexibility, thereby reducing diffusion resistance and enhancing intercellular transport [22,103,118]. Their functional relevance is supported by experimental studies using skin models. For instance, an ethosomal system co-loading curcumin and piperine demonstrated protective effects in human skin explants exposed to environmental stressors, preserving cellular morphology and redox balance, indicating effective cutaneous delivery and biological activity under oxidative stress conditions [69]. Similarly, a quality-by-design optimized resveratrol-loaded ethosomal hydrogel showed enhanced skin permeation and retention, leading to improved antioxidant and anti-photoaging performance compared with non-encapsulated resveratrol in experimental dermatological models [116]. These data indicate that ethanol-mediated lipid perturbation translates into measurable improvements in both cutaneous exposure and functional antioxidant outcomes.

Transferosomes, incorporating edge activators that destabilize the lipid bilayer, are designed to undergo extreme deformation under hydration gradients, enabling passage through intercellular pathways narrower than the vesicle diameter [117,118,119]. Their translational relevance is illustrated by in vivo wound-healing studies: transfersomal formulations containing Centella asiatica actives promoted more efficient excision wound closure in rats, associated with improved dermal delivery of triterpenes and enhanced reparative outcomes compared with conventional systems [120]. These findings support the concept that vesicle deformability can amplify pharmacodynamic responses by improving the delivery of bioactives to viable skin compartments.

Beyond classical ethosomes and transferosomes, other ultradeformable vesicular platforms have been investigated for botanical actives. Rutin-loaded nanovesicles have shown improved physicochemical stability and enhanced topical efficacy of this natural polyphenol, indicating that vesicular encapsulation can convert labile flavonoids into more reproducible dermal actives [25]. Likewise, ultradeformable nanocarriers co-loaded with ammonium glycyrrhizinate and bergamot essential oil demonstrated effective in vivo anti-inflammatory activity, highlighting the ability of flexible vesicles to support delivery and pharmacological performance of complex natural mixtures [26]. Microfluidically engineered ultradeformable liposomes further illustrate how controlled vesicle architecture can be leveraged to optimize skin delivery of plant phytocomplexes [121].

Comparative evidence also indicates that the relative performance of ethosomes and transferosomes depends on payload characteristics and vesicle composition, rather than on a universally superior system. Studies comparing vesicular platforms for phenolic compounds have shown formulation-dependent differences in skin permeability and deposition, underscoring the need for system-specific optimization [119]. Methodologically, such effects are typically demonstrated using ex vivo human skin models, Franz diffusion cells, tape stripping, and microscopy-based biodistribution techniques, which allow quantification of epidermal and dermal localization rather than sole reliance on cumulative permeation [104,105,106].

Collectively, experimental evidence indicates that vesicular systems not only enhance penetration depth but also redefine cutaneous exposure profiles, enabling higher local concentrations of botanical bioactives in viable epidermal and superficial dermal layers. This delivery-driven modulation of tissue compartmentalization is directly linked to improved antioxidant, anti-inflammatory, and reparative responses observed in skin-relevant models [25,26,69,116,120]. Nevertheless, outcomes remain highly formulation-dependent, and cross-study comparisons are often complicated by differences in vesicle composition, elasticity, dosing, and penetration endpoints, highlighting the need for harmonized experimental designs and standardized skin distribution metrics in future translational research [104,105,106,119].

#### 3.3.4. Plant-Derived Extracellular Vesicles as Bioinstructive Delivery Platforms

PDEVs are increasingly recognized as a novel class of bioinstructive nanocarriers that integrate delivery functionality with inherent biological activity, including regenerative and anti-inflammatory effects. Unlike many conventional synthetic nanoparticles, PDEVs possess lipid bilayers, membrane proteins, nucleic acids, and phytochemical cargo that mimic natural intercellular communication systems, enabling not only transport but also direct modulation of cellular processes in recipient cells [27,28,29].

Importantly, recent experimental work has demonstrated that EVs isolated from plant tissues can interact with skin cells via cellular internalization pathways. For example, extracellular vesicles derived from Aloe vera peels were internalized by human keratinocytes (HaCaT) predominantly through clathrin- and caveolae-mediated endocytosis, as evidenced by uptake inhibition assays with pathway-specific inhibitors [122]. Once internalized, these vesicles were shown to exert antioxidant effects, reducing intracellular ROS and upregulating key components of the antioxidant defense system, such as Nrf2 and HO-1 at the transcriptional level in cells under oxidative stress [122].

Beyond modulation of redox balance, PDEVs also influence cellular behaviors linked to tissue repair and regeneration. In vitro scratch assays, a proxy for wound repair potential, revealed that Aloe vera-derived EVs significantly enhance keratinocyte and dermal fibroblast migration, accelerating wound closure in a dose-dependent fashion [122]. Complementary studies using mixed PDEVs from fruit sources showed that treatment of oxidatively damaged fibroblasts not only restored mitochondrial homeostasis but also increased expression of vimentin and matrix metalloproteinase-9 (MMP-9), markers associated with cytoskeletal reorganization and extracellular matrix remodeling during reparative processes [123].

Consistent with a broader regenerative role, evidence from wound healing models indicates that plant EVs can stimulate fibroblast proliferation and migration, enhance endothelial cell functions related to angiogenesis, and promote production of structural proteins such as collagen type I, thereby contributing to multiple facets of cutaneous repair and tissue integrity [124].

These biological outcomes are consistent with the involvement of signaling networks related to cell survival, proliferation, and cytoskeletal dynamics (e.g., PI3K/AKT and MAPK pathways), which are commonly associated with wound repair processes, although the precise molecular intermediates of PDEV action remain under investigation.

#### 3.3.5. Safety-by-Design: Delivery Systems as Determinants of Cutaneous Exposure

Advanced skin delivery systems fundamentally modify the exposure profile of botanical ingredients and must therefore be considered integral determinants of safety, rather than inert formulation components. Physicochemical parameters such as particle size, surface charge, lipid organization, elasticity, and deformability directly influence penetration pathways, cellular uptake, and residence time within specific skin compartments [101,125].

Nanocarriers in the sub-micron range preferentially accumulate within the stratum corneum lipid matrix, follicular reservoirs, and upper viable epidermis, whereas smaller or highly deformable vesicles may access deeper viable layers, potentially intensifying biological responses [103,104]. While this enhanced penetration can improve efficacy, it may also elevate the risk of irritation, sensitization, or systemic exposure, particularly when highly bioactive phytochemicals are involved [126].

The safety-by-design paradigm addresses this challenge by integrating formulation engineering with early toxicological evaluation. Modern strategies include modulation of vesicle composition to restrict deep diffusion, surface functionalization to reduce nonspecific cellular interactions, and controlled-release architectures that prevent concentration spikes in viable tissues [118]. Parallel to formulation optimization, non-animal testing approaches are increasingly implemented at early stages. Reconstructed human epidermis (RHE) models, in vitro irritation and sensitization assays, barrier integrity testing, and advanced penetration assessment techniques allow evaluation of biological impact without reliance on animal models [91,92,127,128].

This integrated approach ensures that delivery systems enhance therapeutic or cosmetic efficacy while maintaining a predictable and controlled exposure profile [85,126,127]. As nanotechnology becomes more prevalent in botanical dermatology, safety-by-design principles are essential for aligning innovation with regulatory expectations and consumer safety [99,126,129].

#### 3.3.6. Translational Impact of Advanced Delivery Systems in Botanical Dermatology

Advanced cutaneous delivery technologies have emerged as critical determinants of the translational performance of botanical actives, as they directly govern molecular stability, penetration kinetics, tissue compartmentalization, and intracellular exposure [103,117,125]. By controlling these parameters, nanostructured and vesicular systems enable predictable modulation of signaling pathways involved in oxidative stress, inflammatory cascades, melanogenesis, and extracellular matrix turnover [16,69,71,110].

Several studies demonstrate that delivery systems substantially alter biological outcomes compared with free phytochemicals. For instance, nanostructured lipid carriers and ethosomal formulations have been shown to enhance dermal retention and antioxidant efficacy of polyphenols such as resveratrol and quercetin, leading to stronger inhibition of ROS generation and matrix metalloproteinase expression in UV-stressed skin models compared to non-encapsulated forms [71,113,114]. Similarly, lipid nanocarriers loaded with curcumin or catechin derivatives enhance anti-inflammatory effects by suppressing NF-κB and MAPK signaling in keratinocytes and fibroblasts [69,110,130].

Translational relevance is particularly evident in wound repair and barrier restoration. Vesicular systems containing Centella asiatica triterpenes, ferulic acid, or botanical antioxidant complexes have demonstrated accelerated re-epithelialization, increased fibroblast proliferation, and improved collagen deposition in in vivo or ex vivo skin models, outcomes that correlate with improved bioavailability and sustained tissue exposure [108,114,120]. Comparable improvements in photoprotection and anti-photoaging efficacy have been observed when carotenoids and flavonoids are delivered through lipid-based nanocarriers, which enhance their stability and prevent oxidative degradation at the skin surface [60,68,71].

Collectively, these findings indicate that advanced delivery systems do not merely enhance penetration but reshape the pharmacodynamic profile of botanical compounds [103,125]. By transforming variable, unstable phytochemicals into controlled, bioavailable actives, these technologies effectively bridge the gap between in vitro bioactivity and in vivo efficacy [107,125]. Their rational integration is therefore a prerequisite for the development of reproducible, safe, and clinically translatable botanical dermatological formulations.

## 4. Dermatological Applications of Botanical-Based Products

The increasing demand for safer, sustainable, and multifunctional skincare solutions has promoted the integration of botanical ingredients into dermatological practice. However, the strength of scientific evidence supporting their use varies considerably across different skin conditions and experimental models. To provide a critical and translation perspective, this section organizes dermatological applications of botanical-based products according to the level of available evidence, distinguishing between preclinical in vitro and in vivo studies and human clinical investigations. This approach allows a clearer assessment of therapeutic potential, translation gaps, and clinical relevance. Importantly, the clinical performance of many botanical actives is strongly influenced by formulation design and delivery strategy, as discussed in Section 3. Advanced carriers, including lipid nanoparticles and vesicular systems, have been shown to improve stability, dermal bioavailability, and therapeutic performance of several phytochemicals.

### 4.1. Anti-Aging and Anti-Wrinkle Effects

Skin aging results from the interplay between intrinsic factors, including chronological aging, genetic background, and extrinsic stressors such as ultraviolet radiation and environmental pollution. These processes converge on oxidative stress generation, ECM degradation, and progressive loss of skin elasticity. Preclinical studies in keratinocytes and dermal fibroblasts demonstrate that botanical polyphenols, flavonoids, and terpenoids can modulate key pathways involved in skin aging by enhancing collagen synthesis, inhibiting elastase and hyaluronidase activity, and suppressing MMP expression, under UV or ROS-induced stress conditions [70,97]. At the molecular level, select polyphenols such as resveratrol, EGCG, and quercetin have been shown to attenuate cellular senescence markers in skin-relevant models through activation of longevity and energy-sensing pathways, including SIRT1 and AMP-activated protein kinase (AMPK) signaling [57,98]. These mechanisms are associated with improved mitochondrial function, enhanced stress resistance, and preservation of fibroblast activity, providing a mechanistic rationale for their anti-aging effects. Beyond polyphenols, terpenoid compounds also contribute to anti-aging strategies. Bakuchiol, a meroterpene from *Psoralea corylifolia*, has been reported in in vitro and clinical studies to promote fibroblast proliferation and collagen deposition through retinol-like mechanisms, while exhibiting a more favorable tolerability profile compared with conventional retinoids [131].

Similarly, extracts from *Centella asiatica* enhance skin density and hydration by activating transforming growth factor-β (TGF-β) dependent pathways that stimulate fibroblast proliferation and ECM synthesis, as demonstrated in both experimental models and human studies [120,132]. Importantly, clinical investigations of topical formulations enriched with polyphenols or *Centella asiatica* extracts report reductions in wrinkle depth and improvements in skin firmness following long-term application [133,134]. However, while these findings support the translational potential of botanical-based anti-aging products, the magnitude of clinical benefit varies across formulations and study designs, underscoring the need for well-controlled trials to define efficacy, optimal dosing, and duration of treatment. Notably, many of these outcomes have been shown to depend strongly on formulation and delivery strategy, as insufficient cutaneous bioavailability remains a limiting factor for several polyphenols and terpenoids

### 4.2. Photoprotection and Anti-Pigmentation

Botanical compounds contribute to skin photoprotection and pigmentation control through complementary but mechanistically distinct pathways. Photoprotective effects are primarily associated with the attenuation of UV-induced oxidative stress and inflammation, whereas anti-pigmentation activity involves the modulation of melanocyte signaling and melanin biosynthesis. Polyphenols from green tea, grape seed, and pomegranate have been shown in keratinocyte and fibroblast-based models to reduce ultraviolet-induced ROS production and suppress pro-inflammatory mediators such as COX-2 and tumor necrosis factor-α (TNF-α). These effects contribute to the mitigation of UV-driven cellular damage and provide an indirect protective environment against photoinduced hyperpigmentation [135,136,137,138]. Direct regulation of melanogenesis is mediated by specific botanical constituents that target key enzymatic and transcriptional regulators of pigment production. Glabridin (licorice), arbutin (bearberry), and ellagic acid (berries) have been reported to inhibit tyrosinase activity and downregulate melanin biosynthesis in melanocyte models, resulting in reduced pigmentation in preclinical and selected clinical studies [139,140,141,142]. Additional compounds, such as aloesin, interfere with melanocyte-stimulating signaling pathways, further supporting their depigmenting potential. Although not of botanical origin, niacinamide is frequently combined with plant-derived actives in cosmetic formulations due to its complementary ability to modulate melanosome transfer and enhance barrier function, thereby improving overall depigmenting efficacy [143].

Beyond pigmentation control, select botanical metabolites contribute to dermal resilience and photoaging prevention. Isoflavones such as genistein and daidzein act as phytoestrogens and have been shown to stimulate collagen synthesis and improving dermal thickness in skin models, with clinical relevance reported particularly in post-menopausal populations [143]. Anthocyanins, abundant in berries and grapes, exhibite strong ROS scavenging and anti-inflammatory properties and have been associated with reinforcement of the epidermal barrier, supporting their role in protecting against photoinduced damage and pigmentary alteration [144]. Collectively, these findings indicate that botanical-based photoprotective and depigmenting strategies operate through multi-level mechanisms involving oxidative stress control, inflammatory modulation, and direct regulation of melanocyte function. However, variability in formulation, dosage, and study design highlights the need for well-controlled clinical trials to better define efficacy and long-term safety. Encapsulation strategies have been shown to enhance stability and skin retention of these compounds, which is critical for sustained photoprotective efficacy.

### 4.3. Wound Healing and Skin Regeneration

Phytochemicals have been shown to influence multiple phases of the wound healing process, including hemostasis, inflammation, proliferation, and tissue remodeling. Preclinical studies using fibroblast, keratinocytes, and in vivo wound models indicate that polysaccharides from *Aloe vera* and oat-derived β-glucans enhance fibroblast proliferation, promote keratinocyte migration, and stimulate angiogenesis through the upregulation of growth factors such as vascular endothelial growth factor (VEGF) and fibroblast growth factor (FGF) [145,146,147]. These effects contribute to accelerated granulation tissue formation and early wound closure in experimental setting. Triterpenes from *Centella asiatica* have been extensively investigated for their regenerative properties and have been shown to stimulate collagen synthesis, angiogenesis, and re-epithelialization by modulating TGF-β dependent pathways [148,149]. Similarly, propolis and selected plant-derived terpenes demonstrate anti-inflammatory activity and support ECM remodeling by regulating cytokine release and fibroblast function during the proliferative and remodeling phases of wound repair [150,151,152].

While extensive preclinical evidence supports the wound-healing activity of botanical-based formulations, clinical data remain comparatively heterogeneous. Human studies have reported improvements in wound closure kinetics, scar quality, and biomechanical properties of regenerated skin following topical application of selected plant-derived preparations. However, clinical outcomes are strongly influenced by formulation design, bioactive concentration, wound etiology, and study methodology [149]. These factors highlight the need for standardized formulations, well-controlled randomized clinical trials, and harmonized outcome measures to establish reproducible efficacy and safety profiles [132].

### 4.4. Anti-Inflammatory and Antimicrobial Effects

Botanical compounds exert anti-inflammatory effects by modulating key signaling pathways involved in cutaneous immune responses and chronic inflammation. In skin-relevant cellular models, several phytochemicals have been shown to attenuate the activation of NF-κB and downregulate the expression of pro-inflammatory mediators, including COX-2 and iNOS, resulting in reduced cytokine release and oxidative stress in keratinocytes and dermal fibroblasts [130,153,154]. Curcumin, apigenin, and luteolin represent well-characterized examples of compounds capable of suppressing inflammatory cascades under oxidative or cytokine induced stress conditions. In addition to immunomodulatory activity, several botanical metabolites display antimicrobial properties that are particularly relevant for inflammatory skin disorders with a microbial component. Terpenes, flavonoids, and selected alkaloids have demonstrated inhibitory activity against common cutaneous pathogens, including *Staphylococcus aureus* and *Cutibacterium acnes* (formerly *Propionibacterium acnes*), in in vitro antimicrobial assays and skin infection models [155,156,157,158]. By reducing microbial load while simultaneously attenuating inflammatory signaling, these compounds may provide dual benefits in conditions such as acne, infected wounds, and inflammatory dermatoses.

Kaempferol, a flavonol present in multiple plant species, exhibits broad antimicrobial and anti-inflammatory effects. Experimental studies indicate that kaempferol suppresses pro-inflammatory cytokine production and inhibits bacterial growth, supporting its traditional use and contemporary investigation as a multifunctional botanical ingredient for dermatological application [81,159]. However, while preclinical data highlight promising anti-inflammatory and antimicrobial effects, further clinical studies are required to establish optimal formulation, dosing regimens, and long-term safety in human skin.

### 4.5. Acne and Seboregulation

Acne vulgaris is a multifactorial skin disorder involving dysregulated sebum production, follicular hyperkeratinization, microbial colonization, and inflammatory responses [160]. Botanical compounds have been investigated as complementary approaches to acne management due to their ability to target multiple pathogenic mechanisms simultaneously [161].

Preclinical and clinical studies indicate that polyphenols derived from green tea, particularly catechins, can modulate sebaceous gland activity by inhibiting 5α-reductase, thereby reducing sebum production and contributing to improvements in acne lesion severity [162,163]. These effects are complemented by anti-inflammatory activity, which further supports their relevance in acne-prone skin. In parallel, several botanical ingredients exhibit antimicrobial activity against acne-associated microorganisms [164]. Tea tree oil, curcumin, and selected phenolic acids have been shown in in vitro assays and clinical studies to suppress the growth of *Cutibacterium acnes* and other skin-associated bacteria, while also attenuating local inflammatory responses [164]. Clinical investigations report reductions in inflammatory lesion counts and improvements in acne severity following topical application of formulations containing these botanical actives [165,166,167,168]. Despite promising results, the clinical efficacy of botanical-based acne treatments remains influenced by formulation, concentration, treatment duration, and individual skin sensitivity. In particular, essential oils require careful dosing and safety assessment to minimize irritation and sensitization, highlighting the importance of standardized formulations and controlled clinical evaluation.

### 4.6. Atopic Dermatitis and Barrier Repair

Atopic dermatitis is a chronic inflammatory skin disorder characterized by impaired epidermal barrier function, increased transepidermal water loss (TEWL), immune dysregulation, and alterations. Botanical-based interventions have been explored as supportive strategies to address these interconnected pathogenic components [3,4].

Botanical oils rich in linoleic acid (e.g., evening primrose, borage, sunflower) have been shown in clinical and experimental studies to improve the lipid profile of the stratum corneum and reduce TEWL in atopic and xerotic skin. These effects are mechanistically linked to the replenishment of essential fatty acids required for ceramide synthesis and epidermal lipid organization [169]. In parallel, flavonoid-rich extracts from chamomile and licorice exert soothing and anti-pruritic effects by modulating mast cell-derived histamine release and downregulating key pro-inflammatory cytokines implicated in atopic skin inflammation [170]. In skin-relevant models and clinical observations, these botanicals have been associated with reduced erythema and symptomatic relief, supporting their role in managing inflammatory flares rather than as primary therapeutic agents [171,172,173].

Overall, botanical-based approaches may provide adjunctive benefits in atopic dermatitis by targeting barrier dysfunction, inflammation, and microbial imbalance. Nevertheless, given the chronic nature of the disease and the variability in individual sensitivity, standardized formulations, careful safety assessment, and well-designed clinical trials are required to define their role within evidence-based management strategies.

### 4.7. Clinical and Cosmeceutical Implications

Botanical-based dermocosmetic and cosmeceutical formulations have gained increasing attention due to growing clinical interest in multifunctional, skin-compatible products [4,174,175]. A body of clinical and observational studies suggests that standardized botanical extracts, including *Centella asiatica*, *Licorice*, *Aloe vera*, and polyphenol-rich complexes, are associated with improvements in parameters such as skin hydration, elasticity, and overall skin appearance when incorporated into topical formulations [4,155,176,177].

The clinical relevance of these effects is largely attributed to the pleiotropic activity of botanical compounds, which simultaneously target oxidative stress, inflammation, barrier function, and extracellular matrix remodeling [178,179,180]. This multifunctionality, combined with generally favorable tolerability profiles when appropriately formulated, supports their use in dermocosmetic products intended for sensitive skin and adjunctive post-procedural care [181]. However, the magnitude and reproducibility of clinical outcomes vary across studies, reflecting differences in formulation composition, active concentration, study design, and treatment duration [180,182,183].

Despite increasing research activity, the number of well-designed randomized clinical trials evaluating botanical dermatological formulations remains relatively limited. Consequently, registry-based evidence should be interpreted cautiously and primarily reflects exploratory clinical interest rather than consolidated therapeutic validation.

Several clinical studies are ongoing or have been completed. In this context, registered clinical investigations provide an important translational bridge between experimental evidence and real-world cosmeceutical application. As summarized in Table 1, several interventional studies registered on ClinicalTrials.gov have explored the impact of botanical-derived or nutraceutical formulations on skin and appendage health in healthy volunteers. These studies primarily involve oral or topical supplementation strategies based on polyphenols, standardized botanical extracts, biotin-containing plant complexes, and multi-ingredient nutraceutical formulations.

For example, trial NCT03487965 evaluated the effects of low- and high-dose polyphenol dietary supplementation on skin attributes in adult female volunteers, reflecting the growing interest in systemic antioxidant strategies for improving cutaneous appearance and barrier function. Similarly, NCT05972512 investigated standardized botanical biotin extracts, alone or combined with silica, targeting hair and skin quality parameters, consistent with the recognized role of micronutrient-plant combinations in integumentary support.

Studies such as NCT05332743, assessing a plant-based nutraceutical formulation designed for female hair health, further highlight the expansion of cosmeceutical research toward orally administered botanical complexes with potential effects on follicular biology and scalp condition. In parallel, innovative approaches are emerging, as illustrated by NCT06930326, which explored intradermal administration of exosome-based preparations compared with saline control, representing a convergence between botanical bioactives, extracellular vesicle research, and aesthetic dermatology. Additional trials, including NCT06097871 and the ongoing NCT06841458, focus on multi-component skin nutraceutical supplements in young to middle-aged adults, reflecting continued clinical interest in preventive and appearance-oriented dermocosmetic strategies.

However, despite the growing number of registered studies, many of these trials do not yet have corresponding peer-reviewed publications reporting detailed outcomes. Consequently, while registry data confirm the feasibility and safety orientation of such interventions, the magnitude of clinical benefit, optimal dosing, and long-term efficacy remain insufficiently standardized. This gap underscores the need for well-designed, adequately powered, placebo-controlled trials with harmonized endpoints (e.g., instrumental measures of hydration, elasticity, transepidermal water loss, and imaging-based wrinkle analysis) to strengthen the evidence base for botanical cosmeceuticals.

Although these registered trials indicate growing clinical interest in botanical and nutraceutical dermatology, many studies lack published peer-reviewed outcomes, and their design, endpoints, and formulations are heterogeneous. Therefore, these data should be interpreted as indicators of translational exploration rather than consolidated clinical evidence.

## 5. Sustainability in Natural Cosmetics: Circular Economy and Green Innovation

The rapid growth of the natural cosmetics industry has spurred increasing attention to sustainability, not only in terms of ingredient origin but across the entire life cycle of cosmetic products. The concept of sustainability in dermatological cosmetics encompasses ethical sourcing, environmentally responsible manufacturing processes, biodegradable packaging, and the reduction in environmental impact post-consumption. This comprehensive approach aligns with global goals of sustainable development and the principles of the circular economy, emphasizing resource efficiency, waste minimization, and environmental protection. In the context of dermatological pharmaceutics, sustainability considerations increasingly intersect with formulation science, as raw material sourcing, extraction technologies, and packaging choices directly influence the safety, reproducibility, and overall life-cycle performance of botanical-based delivery systems.

### 5.1. Life Cycle Assessment and Environmental Impact

A key methodology for evaluating sustainability in cosmetics LCA, which quantifies the environmental impact associated with every stage of a product’s life, from raw material extraction and production to distribution, use, and disposal. In the context of natural cosmetics, LCA is instrumental in identifying hotspots in the supply chain, such as water and energy consumption during plant cultivation and extraction, emissions during formulation and packaging, and waste generation during usage and disposal [9]. LCA enables formulation scientists and sustainability officers to optimize production strategies and transition toward lower-impact alternatives, such as waterless formulations, cold-processing techniques, and the use of solar or renewable energy sources during manufacturing [185,186].

### 5.2. Upcycling of Botanical By-Products

An emerging and impactful strategy within sustainable cosmetics is upcycling, which involves transforming agro-industrial waste or by-products into valuable cosmetic ingredients [187]. This approach not only reduces environmental burden but also enhances the economic value of botanical biomass. For instance, fruit peels, seeds, and pomace from the food and wine industries are rich in bioactive compounds such as flavonoids, phenolic acids, carotenoids, and unsaturated fatty acids. These waste-derived extracts have demonstrated antioxidant, anti-inflammatory, and anti-aging properties suitable for dermatological applications.

Common examples include:

❖ Citrus peels (e.g., orange, lemon, bergamot): a rich source of vitamin C, flavanones, and essential oils with brightening and antioxidant activity [188].
❖ Olive pomace and leaves: high in hydroxytyrosol, oleuropein, and squalene, offering moisturizing and anti-aging effects [189,190,191].
❖ Tomato skins and seeds: rich in lycopene and unsaturated fatty acids, used for photoprotection and skin repair [55,192,193].
❖ Grape pomace (*Vitis vinifera*): a potent source of resveratrol and anthocyanins, with anti-aging and anti-inflammatory properties [194,195].

**Figure 2:**
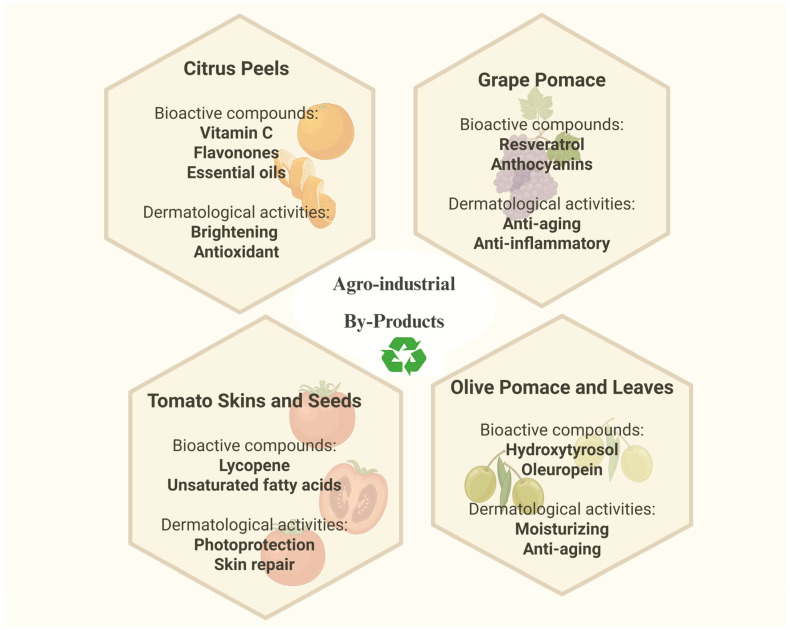
Upcycled agro-industrial by-products as sustainable sources of bioactive compounds for dermatological formulations. Agro-industrial residues such as citrus peels, grape pomace, tomato skins and seeds, and olive pomace and leaves represent valuable sources of phytochemicals, including flavanones, polyphenols, carotenoids, and olive-derived phenolic compounds such as hydroxytyrosol and oleuropein. Through green extraction and valorization strategies, these compounds can be recovered from agricultural waste streams and incorporated into dermatological formulations. Once formulated, these bioactives can exert multiple beneficial activities on the skin, including antioxidant, anti-inflammatory, photoprotective, moisturizing, and skin-repair effects, supporting the development of sustainable and circular cosmetic and dermatological products. Created in BioRender. Cristiano, M. C. (2026). https://BioRender.com/firmmg3, accessed on 11 March 2026.

The use of these by-products contributes to circular economy models and addresses consumer demand for clean, waste-free beauty products (Figure 2).

### 5.3. Sustainable Packaging and Biodegradable Materials

Although packaging does not directly influence pharmacodynamics, it represents a major determinant of overall product life-cycle sustainability. Packaging remains a critical concern in the cosmetics industry, with a growing shift toward recyclable, compostable, and refillable formats. Innovations include the use of bioplastics derived from sugarcane, corn starch, and cellulose; glass and aluminum containers with high recyclability, and refill stations to minimize single-use packaging [196,197]. Brands are increasingly adopting life-cycle thinking in their packaging design, including carbon footprint labeling and take-back programs for used containers [198,199,200].

### 5.4. Green Chemistry and Low-Impact Extraction Technologies

To further align with environmental goals, the development of cosmetic ingredients increasingly follows green chemistry principles. These include closed loop operating systems, solvent-free or low-toxicity extraction techniques, such as supercritical CO_2_ extraction, microwave-assisted extraction, and pressurized hot water extraction, which reduce energy usage and avoid hazardous reagents [95,96,121]. These methods preserve the integrity of sensitive phytocompounds, improve extraction yields, and minimize chemical waste [95,96,121]. In parallel, the replacement of petrochemical solvents with bio-based solvents derived from fermentation or plant oils reflects a broader industry shift toward eco-responsibility [201].

### 5.5. Consumer Education and Transparent Labeling

A crucial but often overlooked aspect of sustainability in natural cosmetics is consumer behavior. In response, cosmetic brands are increasingly investing in transparent communication strategies to educate consumers on ingredient sourcing, packaging disposal, and product longevity. This consumer-facing dimension of sustainability plays a key role in translating environmental commitments into responsible purchasing and usage practices. Certification labels and standards such as COSMOS, ECOCERT, and NATRUE are widely used in this context to provide standardized criteria for natural and organic cosmetics. These labels do not act as regulatory authorities but function as reference frameworks that support transparency comparability, and consumer trust, thereby helping to reduce misleading sustainability claims and greenwashing practices [202]. In parallel, digital tools designed for consumer engagement are gaining relevance QR-code-enabled traceability systems, blockchain-based authentication, and digital environmental scoring schemes allow consumers to access information on ingredient origin, supply chain practices, and environmental impact directly at the point of purchase. By improving accessibility to sustainability-related data, these tools promote informed decision-making and greater accountability throughout the product life cycle [203]. Overall, engagement represents an essential component of sustainable cosmetic development, completing formulation and manufacturing strategies and reinforcing the role of end users in reducing the environmental footprint of cosmetic products (Table 2).

### 5.6. Industrial Perspectives and Alignment with Sustainable Development Goals (SDGs)

From an industrial and policy perspective, sustainability in the cosmetics industry is increasingly aligned with global frameworks such as the United Nations Sustainable Development Goals (SDGs). Cosmetic manufacturers are progressively integrating circular economy models and eco-design principles that emphasize resource efficiency, biodiversity protection, and reduced environmental footprints across production systems [9,216,217]. These strategies not only mitigate environmental impact but also support SDG objectives such as responsible consumption and production, climate action, and sustainable innovation.

From an industrial perspective, sustainability has evolved from a niche concept into a key driver of competitiveness and long-term brand value. Leading companies now incorporate LCAs, renewable energy, and waste valorization into their production pipelines to quantify and reduce environmental impact [218,219]. Such practices enable data-driven decision-making and support the transition toward low-impact manufacturing models. Voluntary certification standards, including COSMOS, ECOCERT, and NATRUE, also play an important role at the industrial level by providing harmonized criteria for natural and organic cosmetic products. While not regulatory bodies, these schemes help validate sustainability-related claims, facilitate market differentiation, and reduce the risk of greenwashing when combined with internal quality control systems and regulatory compliance [202]. In parallel, digital innovation is reshaping industrial sustainability management. Technologies such as blockchain-based supply chain tracking, digital traceability platforms, and environmental performance metrics are increasingly adopted to enhance transparency, traceability, and accountability across complex global supply networks [203]. These tools support both internal sustainability governance and external reporting requirements.

Overall, aligning natural and sustainable cosmetics with SDGs underscores their sector’s potential to serve as models of responsible industrial practice. By integrating scientific innovation, environmental assessment, digital governance, and regulatory compliance, the cosmetics industry is positioned to contribute meaningfully to the transition toward a more ethical, resilient, and environmentally responsible dermatological future.

### 5.7. Challenges and Future Perspectives

Despite recent advances in natural and sustainable cosmetics, several scientific, technical, and regulatory challenges persist. The standardization of sustainability metrics, particularly for upcycled ingredients, is still inconsistent, and certification costs or technological limitations may restrict accessibility for smaller producers. Furthermore, the sourcing of exotic or rare botanicals raises concerns regarding biodiversity loss, ecosystem disruption, and long-term supply sustainability, emphasizing the need for responsible sourcing strategies and alternative production models.

**Figure 3:**
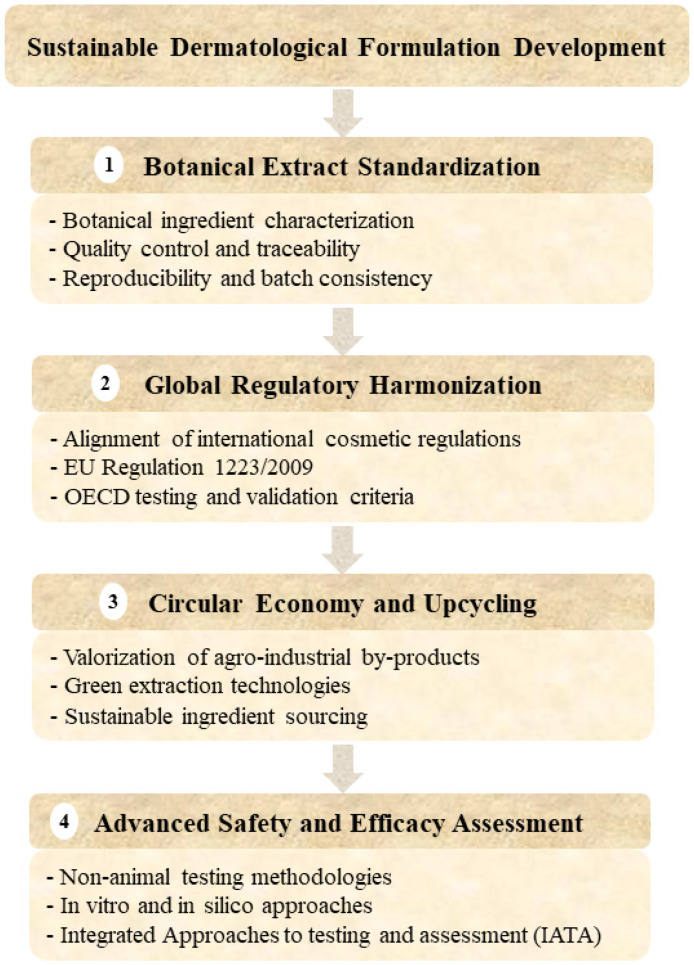
Conceptual framework supporting sustainable dermatological formulation development based on botanical ingredients. The schematic overview illustrates four interconnected pillars that guide the development of sustainable dermatological formulations derived from botanical ingredients. These include botanical extract standardization, ensuring ingredient characterization, quality control, traceability, and batch-to-batch reproducibility; global regulatory harmonization, involving the alignment of international cosmetic regulations, including EU Regulation 1223/2009 and OECD testing guidelines; circular economy strategies based on the upcycling and valorization of agro-industrial by-products through green extraction technologies and sustainable sourcing; and advanced safety and efficacy assessment, which incorporates alternative non-animal methodologies, in vitro and in silico approaches, and IATA. Together, these elements provide a multidisciplinary framework that supports the development of safe, reproducible, and pharmaceutically relevant dermocosmetic formulations.

Looking ahead, future developments in sustainable dermatological cosmetics are expected to emerge from the convergence of digital technologies, biotechnology, and regulatory innovation. Key research and industrial directions include the application of artificial intelligence (AI) and blockchain-based systems to optimize formulation design, monitor sustainability indicators, and enhance supply chain transparency. At the ingredient level, increasing attention is being directed toward microbiome-friendly compounds, such as prebiotics and post biotics derived from renewable sources, to balance skin health with ecological compatibility. In parallel, biosynthetic alternatives produced through plant cell culture or microbial fermentation are gaining relevance as potential substitutes for rare or overharvested botanical species, offering improved reproducibility and reduced environmental pressure. Finally, regulatory harmonization across international markets represents a critical step toward defining robust sustainability claims, preventing greenwashing practices, and encouraging the adoption of standardized eco-certification frameworks. Collectively, these perspectives highlight the need to balance innovation with responsibility ensuring that the future of sustainable dermatology is grounded in scientific evidence, environmental stewardship, and transparent governance (Figure 3).

## 6. Regulatory and Safety Aspects of Natural Cosmetics

The global demand for green, sustainable, and natural cosmetics has catalyzed a significant shift in regulatory priorities, prompting authorities and industry stakeholders to redefine safety, quality, and marketing standards. While products labeled as “natural” are often perceived by consumers as inherently safe and environmentally friendly, scientific evidence and regulatory experience clearly indicate that natural origin alone does not guarantee safety or tolerability, particularly in the context of complex botanical mixtures, concentrated extracts, and innovative delivery systems [85,86,87,100,126].

The absence of harmonized legal definitions of “natural” cosmetics, combined with substantial differences in regulatory approaches across regions, poses a major challenge for both manufacturers and regulators. Variability in safety assessment requirements, toxicological testing strategies, labeling rules, and claims substantiation complicates product development, international trade, and consumer protection [85,99,100,202]. At the same time, this fragmented regulatory landscape offers opportunities to strengthen international convergence, promote evidence-based safety evaluation, and improve transparency and consistency in sustainability-related claims, as emphasized by international regulatory cooperation initiatives [36,93,99,202].

In this context, regulatory oversight of natural cosmetics must balance innovation with rigorous safety assessment, addressing not only ingredient origin but also toxicological risk, formulation complexity, potential contaminants, and long-term consumer exposure. The following sections examine key regulatory definitions, certification schemes, regional regulatory divergences, safety and toxicological evaluation strategies, contaminant control, labeling requirements, and emerging efforts toward global harmonization in the natural cosmetics sector [93,99].

### 6.1. Definition and Classification of Natural Cosmetics

The term “natural cosmetics” lacks a universally accepted legal definition, which complicates labeling, market positioning, and regulatory compliance. Within the European Union (EU), cosmetic products are governed under the general framework of Regulation (EC) No. 1223/2009, which establishes comprehensive requirements for safety assessments, prohibited ingredients, labeling, and good manufacturing practices [99,202]. However, this regulatory framework does not define or recognize “natural” or “organic” cosmetics as distinct legal categories, treating all cosmetic products under the same safety and compliance obligations regardless of ingredient origin [99].

In the absence of a legally binding definition, a number of voluntary private certification standards have been developed to address consumer demand for natural and organic cosmetic production [202]. Certification schemes such as COSMOS, NATRUE, and ECOCERT provide standardized criteria for the classification of cosmetics based on ingredient origin, processing method, and environmental considerations. These standards typically include requirements related to use of renewable raw materials, limitations on chemical transformation processes, and restrictions on petrochemical derived ingredient, microplastics, and certain synthetic substances [7,99,202]. For instance, the COSMOS standard establishes minimum thresholds of natural and organic content and specifies permitted and prohibited ingredients according to sustainability and biodegradability criteria. While these certification schemes do not carry legal authority, they play a significant role in harmonizing industry practices, supporting transparency, and guiding both manufacturers and consumers in the interpretation of “natural” and “organic” claims within the cosmetic market [7,202].

### 6.2. Voluntary Certification Standards and Market Transparency

Voluntary third-party certification standards play a significant role in enhancing transparency and consumer trust within the natural cosmetic market. COSMOS and NATRUE establish structured criteria for evaluating cosmetic products with respect to ingredient origin, environmental impact, and sustainability-related practices. Compliance with these standards is typically verified through independent audits conducted by accredited certification organizations, rather than by regulatory authorities [7,202]. Products certified under COSMOS or NATRUE are required to ensure traceability of raw materials, maintain detailed technical documentation, and comply with predefined sustainability criteria, which may include restrictions on certain ingredients, requirements for biodegradable packaging, and environmental performance considerations across the product life cycle. In some cases, certification frameworks also encourage or require the use of LCAs approaches to support sustainability claims and internal quality control processes [218]. In addition, these voluntary certification standards impose specific requirements for the substantiation of marketing claims such as “natural” or “organic,” often requiring quantitative thresholds and documentary evidence to support such statements. Although not legally binding, adherence to recognized certification schemes can provide a competitive market advantage and serve as a reference point for consumers seeking products aligned with ethical, environmental, and sustainability-oriented values [7,202].

### 6.3. Regulatory Divergence Across Regions

Regulatory frameworks governing cosmetic products vary substantially across regions, reflecting different regulatory philosophies, risk assessment approaches, and market oversight models. In the United States, the FDA does not recognize “natural” as a specific legal category, and cosmetics are regulated under the Federal Food, Drug, and Cosmetic Act. This framework relies primarily on post-market surveillance, placing the responsibility for safety substantiation on manufacturers without requiring pre-market authorization, except for color additives [100,220]. As a result, industry led safety assessments and voluntary standards play a particularly prominent role in the United States cosmetic market.

In contrast, several Asian regulatory systems adopt hybrid frameworks incorporating elements of both cosmetic and pharmaceutical regulation, particularly for products positioned between aesthetic and functional/therapeutic claims. In Japan and South Korea, under the supervision of the Ministry of Health, Labour and Welfare and the Ministry (MHLW) of Food and Drug Safety (MFDS), specific categories such as quasi-drugs or functional cosmetics are subject to enhanced regulatory scrutiny, including ingredient pre-approval, efficacy substantiation, and additional safety requirements for functional claims [221]. These frameworks reflect a more precautionary approach, particularly for products positioned at the interface between cosmetic and therapeutic applications. China has recently undergone significant regulatory reform, introducing measures to reduce mandatory animal testing for certain cosmetic products and strengthening post-market surveillance and ingredient registration systems [222]. These changes align with international trends toward alternative testing methods, sustainability, and animal welfare, while maintaining a centralized regulatory oversight structure [99,222]. The lack of regulatory harmonization across major markets poses substantial challenges for the global cosmetic industry. Differences in safety assessment requirements, toxicological testing strategies, and claims substantiation standards necessitate region-specific formulation design, documentation, and marketing approaches [99,222]. In this context, international initiatives such as the International Cooperation on Cosmetics Regulation (ICCR) and the Organization for Economic Cooperation and Development (OECD) play a critical role in promoting convergence of safety principles, non-animal testing strategies, and risk assessment methodologies [99]. Greater alignment among regulatory systems is essential to facilitate innovation, ensure consumer safety, and support the global development of natural and sustainable cosmetic products.

### 6.4. Safety Assessment and Toxicological Evaluation

Although botanical ingredients are often perceived as inherently safe, natural origin does not equate to toxicological safety. Essential oils, plant extracts, and natural preservatives may elicit adverse effects, including allergic contact dermatitis, photosensitivity, or irritant responses [85,86,87]. Therefore, comprehensive safety and toxicological evaluation is essential before market authorization, especially for new botanical actives, nanocarrier-based delivery systems, and innovative formulation technologies [126,127]. These considerations are particularly relevant for advanced delivery systems, which may modify cutaneous penetration and therefore alter exposure and safety profiles. In line with international regulatory expectations the safety assessment of botanical cosmetic ingredients follows a tiered and weight-of-evidence approach increasingly based on Integrated Approaches to IATA. These frameworks combine physicochemical characterization in vitro testing, in silico modeling, and exposure assessment to ensure consumer safety while minimizing animal testing [91,93].

A structured safety evaluation framework typically involves the following components:

1. Phytochemical characterization and purity assessment, involving qualitative and quantitative profiling of active constituents, identification of impurities, and standardization of extract composition to ensure batch-to-batch consistency. This step is essential, as variability in phytochemical profiles may directly influence toxicological behavior and consumer exposure [93].
2. Microbiological and contaminant screening, addressing the presence of pathogenic microorganisms, heavy metals, pesticide residues, mycotoxins, and residual solvents in accordance with internationally accepted safety thresholds. This aspect is particularly critical for botanical cosmetics, where cumulative exposure from repeated topical application may amplify toxicological risk [88,93].
3. In vitro toxicity testing and computational modeling, including cytotoxicity assays on relevant skin cell models (e.g., keratinocytes and dermal fibroblasts), genotoxicity prediction, and in silico toxicological tools aligned with OECD principles. These approaches are increasingly used to map key events within established adverse outcome pathways and support mechanistically informed hazard identification [91,129].
4. Skin irritation and sensitization assessment, relying on validated non-animal methods incorporated into OECD Test Guidelines and cosmetic-specific IATA frameworks. These include reconstructed human epidermis models for skin irritation (OECD TG 439), DPRA (OECD TG 442C), ARE-Nrf2 luciferase assay (OECD TG 442D), h-CLAT (OECD TG 442E), and complementary assays covering protein binding and T-cell–mediated responses (OECD TG 442A/B). Within an IATA framework, these assays are integrated through a weight-of-evidence approach rather than interpreted individually [92,94,128,129].

While the Local Lymph Node Assay (LLNA; OECD TG 429) is no longer permitted for cosmetic ingredients in the European Union, it remains a reference method in regulatory toxicology and continues to inform hazard identification strategies in non-cosmetic regulatory contexts [92,223].

**Figure 4:**
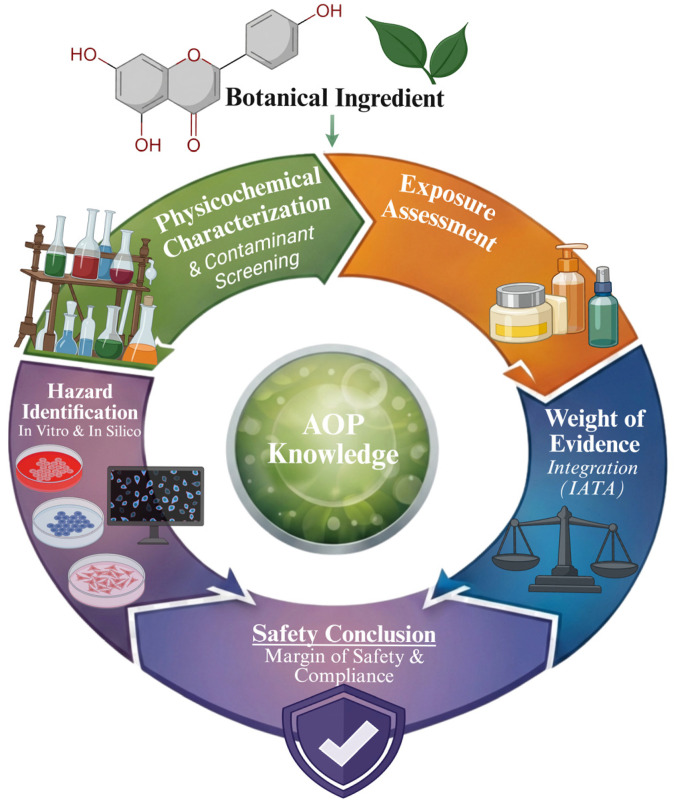
Integrated IATA-based safety assessment framework aligned with OECD Test Guidelines, illustrating the sequential integration of physicochemical characterization, contaminant screening, non-animal in vitro and in silico testing, weight of evidence evaluation, and exposure-driven risk assessment for botanical cosmetic ingredients across regulatory contexts. Created with Created in BioRender. Cristiano, M. C. (2026). https://BioRender.com/6sq78zz, accessed on 29 January 2026.

Overall, the adoption of IATA-based safety assessment strategies, supported by Adverse Outcome Pathway (AOP) knowledge, provides a scientifically robust and ethically responsible approach to evaluating botanical cosmetic ingredients, ensuring regulatory compliance while minimizing the risk of adverse effects in consumers (Figure 4).

### 6.5. Contaminants, Stability, and Shelf-Life

Natural cosmetic formulations are particularly vulnerable to microbial contamination, oxidative degradation, and instability due to the absence of synthetic preservatives and the intrinsic complexity of botanical matrices and frequent reduction or exclusion of conventional synthetic preservatives. The high content of water, sugar, polysaccharides, and unsaturated lipids in plant-derived ingredients creates favorable conditions for microbial growth and oxidative reactions, potentially compromising both product safety and efficacy during storage and use [224,225]. To mitigate these vulnerabilities, manufacturers implement accelerated stability testing, package compatibility assessments, and formulation optimization strategies, including pH control and antioxidant systems, to ensure acceptable shelf-life and product performance [226,227].

Beyond formulation-related instability, raw botanical materials represent a critical control point within the cosmetic supply chain and require rigorous contaminant screening. Environmental exposure, agricultural practices, and post-harvest processing can result in contamination with heavy metals (e.g., arsenic, lead, cadmium), pesticide residues, polycyclic aromatic hydrocarbons, and mycotoxins such as aflatoxins, all of which pose potential toxicological risks even at low concentrations [89,90]. These concerns are particularly relevant for natural cosmetics, which are often applied repeatedly over long periods and, in some cases, on compromised or inflamed skin, increasing the relevance of cumulative exposure and mixture toxicity. Consequently, safety evaluation of botanical ingredients extends beyond intrinsic ingredient toxicity to include systematic assessment of contaminants, batch-to-batch variability, and long-term stability. International regulatory and advisory bodies, including the Scientific Committee on Consumer Safety (SCCS) and the World Health Organization (WHO), emphasize the need for robust analytical quality control, adherence to established safety thresholds, and exposure-based risk assessment approaches when evaluating plant-derived cosmetic ingredients [88,93]. Compliance with these frameworks is essential not only to ensure consumer safety but also to support regulatory acceptance and marketability of natural cosmetic products.

### 6.6. Labeling and Claims Substantiation

Accurate and transparent labeling represents a central pillar of regulatory compliance and consumer trust in the natural cosmetics sector. Beyond fulfilling legal obligations, labeling directly influences consumer perception of safety, efficacy, and sustainability. Consequently, the use of vague or absolute terms such as “chemical-free” or “non-toxic” or “100% safe” is scientifically unfounded and increasingly regarded as misleading or deceptive under multiple regulatory frameworks, particularly when such claims cannot be substantiated by robust toxicological or clinical evidence. Claims commonly associated with natural cosmetics, such as “hypoallergenic,” or “organic,” “suitable for sensitive skin” or “dermatologically tested” require appropriate scientific justification proportional to the nature of the claim. In practice, this includes well designed clinical studies, dermatological tolerance tests, or human repeat insult patch tests (HRIPT), supported by exposure based safety assessment and relevant in vitro or in silico data where applicable [99]. Importantly, the growing complexity of botanical formulations and delivery systems further reinforces the need for claim substantiation based on the final formulation, rather than on individual ingredients alone.

Within the European Union, Commission Regulation (EU) No. 655/2013 establishes harmonized criteria for cosmetic claims, including legal compliance, evidential support, and fair communication [228]. Under this framework, manufacturers are required to maintain comprehensive technical documentation demonstrating that claim is supported by adequate and reproducible evidence, aligned with the product’s actual performance and safety profile [229]. Failure to comply may result in regulatory enforcement actions, market withdrawal, or reputational damage. From a scientific and regulatory perspective, claim substantiation also plays a critical role in preventing greenwashing and ensuring fair competition. As sustainability-related claims (e.g., “eco-friendly,” “natural,” “sustainably sourced”) continue to proliferate, regulatory authorities increasingly scrutinize whether such statements are supported by verifiable data, such as life cycle assessments, certified sourcing schemes, or traceability documentation [35,36,202,215,218]. In this context, rigorous claim substantiation is not merely a compliance exercise but an essential component of responsible innovation and transparent communication in natural and sustainable cosmetics.

### 6.7. Future Perspectives and Global Harmonization

The continued growth of the green cosmetic market highlights the need for greater regulatory harmonization across major global jurisdictions. Differences in safety assessment strategies, testing requirements, and claims substantiation criteria currently represent a major barrier to innovation and international market access. In this context, international initiatives such as the International Cooperation on Cosmetics Regulation (ICCR) play a central role in promoting convergence of regulatory principles, with a specific focus on safety assessment methodologies, non-animal testing strategies, and risk-based decision-making framework [99,230]. A key driver of regulatory alignment is the progressive adoption of IATA, developed under the auspices of the OECD [231,232]. These approaches integrate physicochemical characterization, in vitro and in silico methods, and weight of evidence strategies to support hazard identification and risk assessment while reducing reliance on animal testing. Recent OECD guidance documents and updated Test Guidelines for skin irritation and sensitization (including OECD TG 439, 442C, 442D, and 442E) have provided a scientifically robust and internationally accepted framework that is increasingly referenced by regulatory authorities beyond the European Union, including the United States and several Asian markets [233,234]. Parallel to advances in toxicological science, digital innovation is expected to play an increasingly important role in regulatory compliance and supply chain governance [235]. Blockchain-based traceability systems have been proposed as tools to improve transparency, authenticity verification, and raw material traceability in complex botanical supply chains, while artificial intelligence-driven platforms are being explored to support safety data analysis, ingredient screening, and regulatory decision support [235,236]. Although still at an early stage of implementation, these technologies align with regulatory and consumer demands for transparency and data-driven substantiation of safety and sustainability claims [237].

Looking ahead, the integration of sustainability metrics into regulatory evaluation frameworks represents both an opportunity and a challenge [238]. LCA, carbon footprint analysis, biodegradability, and social impact indicators are increasingly discussed as complementary dimensions of product evaluation, particularly for botanical and upcycled ingredients [239]. However, the lack of standardized methodologies and legally binding thresholds currently limits their consistent regulatory application, highlighting the need for coordinated international guidance to prevent fragmented or misleading sustainability claims [238,239].

In conclusion, the future regulatory evolution of natural cosmetics will depend on the convergence of scientific innovation, regulatory harmonization, and digital transformation. Strengthening international cooperation through ICCR and OECD initiatives, expanding the use of IATA-based non-animal safety assessment strategies, and developing standardized approaches to sustainability evaluation will be essential to support innovation while ensuring consumer safety and environmental responsibility. Such an integrated and globally aligned framework will be critical for the long-term credibility and scientific robustness of natural and sustainable cosmetic products.

## 7. Conclusions and Future Perspectives

The integration of botanical ingredients into modern dermatological formulations reflects a growing alignment between scientific advancement, consumer demand, and sustainability imperatives. Natural cosmetics today are no longer confined to traditional herbal remedies; they represent a dynamic and multidisciplinary field encompassing molecular biology, pharmacognosy, cosmetic technology, and environmental science. The evidence accumulated thus far supports the efficacy of plant-derived bioactives in modulating key biological pathways related to oxidative stress, inflammation, pigmentation, and skin barrier function. However, their effective dermatological translation is not dictated by intrinsic bioactivity alone, but critically depends on formulation design, delivery efficiency, and controlled cutaneous exposure.

From a functional perspective, botanical compounds such as polyphenols, flavonoids, alkaloids, carotenoids, and terpenes exert pleiotropic effects by targeting redox-sensitive and inflammation-related signaling networks, including Nrf2/ARE, NF-kB, MAPKs and SIRT1 dependent pathways. The molecular actions underpin their documented antioxidant, anti-inflammatory, anti-aging, antimicrobial, wound healing, and photoprotective properties. Importantly, the biological efficacy of these compounds is significantly amplified when integrated into advanced skin delivery platforms. Nanoemulsions, SLNs, NLCs, vesicular systems, and emerging bioinstructive carriers such as PDEVs enable improved stability, enhanced epidermal and dermal bioavailability, and spatially controlled delivery, thereby strengthening the exposure-response relationship while minimizing systemic absorption and irritation risk.

Sustainability has emerged as a defining pillar of innovation in botanical dermatology. The valorization of agro-industrial by-products, the application of green chemistry principles, and the promotion of circular economy models are driving the industry toward more ethical, efficient, and eco-friendly practices. The use of upcycled ingredients, waterless formulations, refillable packaging, and biodegradable materials not only minimizes environmental impact but also resonates with the values of a conscious consumer base. This transformation is further reinforced by digital tools, such as blockchain traceability and AI-based formulation optimization, which enhance transparency, safety, and innovation.

Despite this progress, several challenges persist. The complex nature of botanical extracts requires standardized extraction methods, robust quality control, and advanced analytical tools to ensure consistency and reproducibility. Moreover, the expanding use of nanostructured delivery systems requires careful integration of safety-by-design principles, supported by non-animal toxicological testing strategies and internationally harmonized regulatory frameworks. The absence of globally consistent definitions for “natural” and “sustainable” cosmetics continues to complicate claims substantiation and risks undermining consumer confidence through greenwashing practices.

Future perspectives in this field include the exploration of lesser-known plant species, microbiome-friendly botanical formulations, bioengineered phytocompounds, and plant-derived extracellular vesicles. The development of innovative in vitro 3D skin models and in silico predictive tools will further reduce reliance on animal testing and accelerate safety and efficacy assessments. Moreover, collaboration between academia, industry, and policymakers will be crucial to overcoming scientific, technological, and regulatory barriers.

Unlike previous reviews, which have primarily focused on either botanical bioactives, delivery technologies, or sustainability as isolated themes, this work provides an integrated framework that bridges phytochemistry and advanced skin delivery science, regulatory toxicology, and circular economy principles within a dermatological context. The inclusion PDEVs as emerging bioinstructive delivery systems, together with the focus on circular economy strategies such as upcycling and green extraction, highlights underexplored but highly promising directions for dermatological innovation. By explicitly connecting natural cosmetics to global sustainability frameworks and Sustainable Development Goals (SDGs), this review advances the discourse beyond efficacy and safety, situating cosmetic research within a broader context of environmental responsibility, consumer transparency, and industrial transformation.

## Figures and Tables

**Table 1:** Registered clinical studies investigating botanical or nutraceutical interventions for skin and appendage health (ClinicalTrials.gov).

NCT Number	Intervention	Status	Eligible Criteria	Results
NCT03487965	Oral polyphenol dietary supplementation (low dose, high dose) vs. placebo	Completed	Healthy female adults, 30–65 years	No peer-reviewed results published yet (ClinicalTrials.gov record only)
NCT05972512	Standardized botanical biotin extract alone or combined with silica vs. placebo	Completed	Healthy adults, 20–60 years, both sexes	No peer-reviewed results published yet (ClinicalTrials.gov record only)
NCT05332743	Plant-based nutraceutical formulation for female hair health (Nutrafol Vegan Women’s Hair Supplement)	Completed	Healthy male adults, 20–50 years	[184]
NCT06930326	Intradermal exosome preparation vs. 0.9% sodium chloride (saline)	Completed	Healthy male adults, 20–50 years	No peer-reviewed results published yet (ClinicalTrials.gov record only)
NCT06841458	Oral Supplement	Recruiting	Healthy male adults, 18–45 years	No peer-reviewed results published yet (ClinicalTrials.gov record only)
NCT06097871	Skin nutraceutical supplement vs. placebo pill	Completed	Healthy female adults, 18–50 years	No peer-reviewed results published yet (ClinicalTrials.gov record only)

**Table 2:** Overview of sustainable strategies adopted in the cosmetic industry, highlighting environmental benefits, representative examples, and supporting references across the product life cycle.

Approach	Benefits	Examples	References
Green extraction technologies (supercritical CO_2_, ultrasound-assisted, microwave-assisted)	Reduced solvent use, lower energy consumption, preservation of bioactive compounds	Extraction of polyphenols from grape pomace; essential oils via supercritical CO_2_	[204,205]
Upcycling of agro-industrial by-products	Circular economy integration, waste reduction and valorization of natural resources	Citrus peels for antioxidants; olive pomace for squalene; tomato skins for lycopene	[206,207]
Biodegradable and recyclable packaging	Reduced plastic pollution, and environmental footprint	Biopolymer-based containers; recyclable glass or aluminum	[208,209,210]
Waterless or low-water formulations	Conservation of water resources, reduced preservatives	Solid shampoos, concentrated serums, powder cleansers	[185,211]
Renewable energy and eco-design in production	Lower CO_2_ emissions, energy efficiency	Solar-powered manufacturing plants; carbon-neutral facilities	[212,213]
Certification and eco-labeling (COSMOS, ECOCERT, NATRUE)	Consumer trust, transparent communication, standardized sustainability claims	Certified organic creams, eco-labeled sunscreens	[202,214]
Digital transparency and traceability tools (QR codes, blockchain, eco-scores)	Traceability, consumer education, and informed purchasing	Blockchain-authenticated supply chains; QR-coded ingredient sourcing	[203,215]

## Data Availability

No new data were created or analyzed in this study. Data sharing is not applicable to this article.

## References

[1] Deng K., Liu Y., Li D., Liu C. (2025). Applications of Traditional Herbal Ingredients in Skincare: Mapping the Research Landscape and Innovation Trajectories Over Four Decades. J. Cosmet. Dermatol..

[2] Yang J.Y., Guo J.G., Tang P.L., Yan S.D., Wang X.D., Li H.Y., Xie J.L., Deng J.G., Hou X.T., Du Z.C. (2024). Insights from Traditional Chinese Medicine for Restoring Skin Barrier Functions. Pharmaceuticals.

[3] Cizmárová B., Hubková B., Tomecková V., Birková A. (2023). Flavonoids as Promising Natural Compounds in the Prevention and Treatment of Selected Skin Diseases. Int. J. Mol. Sci..

[4] Michalak M. (2023). Plant Extracts as Skin Care and Therapeutic Agents. Int. J. Mol. Sci..

[5] Dini I. (2024). “Edible Beauty”: The Evolution of Environmentally Friendly Cosmetics and Packaging. Antioxidants.

[6] Perret J.K., Velázquez A.G., Mehn A. (2025). Green Cosmetics-The Effects of Package Design on Consumers’ Willingness-to-Pay and Sustainability Perceptions. Sustainability.

[7] Dini I., Laneri S. (2021). The New Challenge of Green Cosmetics: Natural Food Ingredients for Cosmetic Formulations. Molecules.

[8] Ferreira M.S., Magalhaes M.C., Oliveira R., Sousa-Lobo J.M., Almeida I.F. (2021). Trends in the Use of Botanicals in Anti-Aging Cosmetics. Molecules.

[9] Rocca R., Acerbi F., Fumagalli L., Taisch M. (2022). Sustainability paradigm in the cosmetics industry: State of the art. Clean. Waste Syst..

[10] Limbu Y.B., Ahamed A.F.M.J. (2023). What Influences Green Cosmetics Purchase Intention and Behavior? A Systematic Review and Future Research Agenda. Sustainability.

[11] Casale J., Taylor A., Crane J. (2023). Albert M. Kligman, MD (1916–2010): A Controversial Genius in the Field of Dermatology. J. Clin. Aesthetic Dermatol..

[12] Gupta P., Sharma A., Mittal V. (2026). The Beauty Revolution of Nanotechnology: Unveiling the Impact of Cosmetic Nano Wonders. Pharm. Nanotechnol..

[13] Nowak-Perlak M., Olszowy M., Wozniak M. (2025). The Natural Defense: Anti-Aging Potential of Plant-Derived Substances and Technological Solutions Against Photoaging. Int. J. Mol. Sci..

[14] Son D.J., Jung J.C., Choi Y.M., Ryu H.Y., Lee S., Davis B.A. (2020). Wheat Extract Oil (WEO) Attenuates UVB-Induced Photoaging via Collagen Synthesis in Human Keratinocytes and Hairless Mice. Nutrients.

[15] Zhao C., Wu S., Wang H. (2025). Medicinal Plant Extracts Targeting UV-Induced Skin Damage: Molecular Mechanisms and Therapeutic Potential. Int. J. Mol. Sci..

[16] Mancuso A., Cristiano M.C., d’Avanzo N., Panza S., Tarsitano M., Celia C., Paolino D., Fresta M. (2025). Assessing effectiveness of multistage nanomedicines for multidrug therapy of vitiligo. J. Drug Deliv. Sci. Technol..

[17] Boo Y.C. (2022). Ascorbic Acid (Vitamin C) as a Cosmeceutical to Increase Dermal Collagen for Skin Antiaging Purposes: Emerging Combination Therapies. Antioxidants.

[18] Lamie C., Elmowafy E. (2025). Progress in the design of ascorbic acid derivative-mediated drug delivery. RSC Adv..

[19] Harun M.S., Wong T.W., Fong C.W. (2021). Advancing skin delivery of α-tocopherol and γ-tocotrienol for dermatitis treatment via nanotechnology and microwave technology. Int. J. Pharm..

[20] Liu H.-M., Cheng M.-Y., Xun M.-H., Zhao Z.-W., Zhang Y., Tang W., Cheng J., Ni J., Wang W. (2023). Possible Mechanisms of Oxidative Stress-Induced Skin Cellular Senescence, Inflammation, and Cancer and the Therapeutic Potential of Plant Polyphenols. Int. J. Mol. Sci..

[21] Martic I., Jansen-Dürr P., Cavinato M. (2022). Effects of Air Pollution on Cellular Senescence and Skin Aging. Cells.

[22] Akombaetwa N., Ilangala A.B., Thom L., Memvanga P.B. (2023). Current Advances in Lipid Nanosystems Intended for Topical and Transdermal Drug Delivery Applications. Pharmaceutics.

[23] Waghule T., Rapalli V.K., Gorantla S., Saha R.N., Dubey S.K., Puri A., Singhvi G. (2020). Nanostructured Lipid Carriers as Potential Drug Delivery Systems for Skin Disorders. Curr. Pharm. Des..

[24] Yousry C., Saber M.M., Abd-Elsalam W.H. (2022). A Cosmeceutical Topical Water-in-Oil Nanoemulsion of Natural Bioactives: Design of Experiment, in vitro Characterization, and in vivo Skin Performance Against UVB Irradiation-Induced Skin Damages. Int. J. Nanomed..

[25] Cristiano M.C., Barone A., Mancuso A., Torella D., Paolino D. (2021). Rutin-Loaded Nanovesicles for Improved Stability and Enhanced Topical Efficacy of Natural Compound. J. Funct. Biomater..

[26] Cristiano M.C., d’Avanzo N., Mancuso A., Tarsitano M., Barone A., Torella D., Paolino D., Fresta M. (2022). Ammonium glycyrrhizinate and Bergamot essential oil co-loaded ultradeformable nanocarriers: An effective natural nanomedicine for in vivo anti-inflammatory topical therapies. Biomedicines.

[27] Huang D., Chen J., Zhao M., Shen H., Jin Q., Xiao D., Peng Z., Chen T., Zhang Y., Rao D. (2025). Plant-derived extracellular vesicles: Composition, function and clinical potential. J. Transl. Med..

[28] Liu H., Dong T., Dong C., Yang F., Zhou Q., Guan C., Wang W. (2025). Plant-derived exosome-like nanovesicles: A novel therapeutic perspective for skin diseases. J. Nanobiotechnol..

[29] Shao M., Jin X., Chen S., Yang N., Feng G. (2023). Plant-derived extracellular vesicles -a novel clinical anti-inflammatory drug carrier worthy of investigation. Biomed. Pharmacother..

[30] Ligarda-Samanez C.A., Huamán-Carrión M.L., Calsina-Ponce W.C., Cruz G.D.L., Calderón Huamaní D.F., Cabel-Moscoso D.J., Garcia-Espinoza A.J., Sucari-León R., Aroquipa-Durán Y., Muñoz-Saenz J.C. (2025). Technological Innovations and Circular Economy in the Valorization of Agri-Food By-Products: Advances, Challenges and Perspectives. Foods.

[31] Oliveira J.S.d., Oliveira J.P.d., Oliveira C.P.d., Veloso C.M. (2025). Circular economy as a tool for the valorization of agro-industrial residues and the development of biodegradable and sustainable packaging: A bibliometric review from 2015 to 2024. Clean. Circ. Bioecon..

[32] Silva A., Pinto C., Cravo S., Mota S., Rego L., Ratanji S., Quintas C., Silva J.R.E., Afonso C., Tiritan M.E. (2025). Sustainable Skincare Innovation: Cork Powder Extracts as Active Ingredients for Skin Aging. Pharmaceuticals.

[33] Raudone L., Savickiene N. (2024). Phytochemical Profiles of Plant Materials: From Extracts to Added-Value Ingredients. Plants.

[34] Sun S., Yu Y., Jo Y., Han J.H., Xue Y., Cho M., Bae S.J., Ryu D., Park W., Ha K.T. (2025). Impact of extraction techniques on phytochemical composition and bioactivity of natural product mixtures. Front. Pharmacol..

[35] Dwivedi S., Gupta A., Sayal A. (2025). Evolution of consumer perceptions and intentions in the green cosmetics market: A thematic and trend analysis. Front. Sustain..

[36] Lopes J.M., Gomes S., Trancoso T. (2023). The Dark Side of Green Marketing: How Greenwashing Affects Circular Consumption?. Sustainability.

[37] Tu J.-C., Cui Y., Liu L., Yang C. (2024). Perceived Greenwashing and Its Impact on the Green Image of Brands. Sustainability.

[38] Elbouzidi A., Haddou M., Baraich A., Taibi M., El Hachlafi N., Pareek A., Mesnard F., Addi M. (2025). Biochemical insights into specialized plant metabolites: Advancing cosmeceutical applications for skin benefits. J. Agric. Food Res..

[39] Masyita A., Mustika Sari R., Dwi Astuti A., Yasir B., Rahma Rumata N., Emran T.B., Nainu F., Simal-Gandara J. (2022). Terpenes and terpenoids as main bioactive compounds of essential oils, their roles in human health and potential application as natural food preservatives. Food Chem. X.

[40] Riaz M., Khalid R., Afzal M., Anjum F., Fatima H., Zia S., Rasool G., Egbuna C., Mtewa A.G. (2023). Phytobioactive compounds as therapeutic agents for human diseases: A review. Food Sci. Nutr..

[41] Carvalho M.J., Pedrosa S.S., Pintado M., Oliveira A.L.S., Madureira A.R. (2024). New Natural and Sustainable Cosmetic Preservative Based on Sugarcane Straw Extract. Molecules.

[42] Qian H., Shan Y., Gong R., Lin D., Zhang M., Wang C., Wang L. (2022). Mechanism of action and therapeutic effects of oxidative stress and stem cell-based materials in skin aging: Current evidence and future perspectives. Front. Bioeng. Biotechnol..

[43] Wei M., Li M., Li Y., Wang B., Yan Y., Li L. (2025). Upregulation of Receptor Interacting Protein 1 Induced by UVB Contributes to Photodamage of the Skin Through NF-κB Pathway In Vivo and In Vitro. J. Cosmet. Dermatol..

[44] Chaiprasongsuk A., Lohakul J., Soontrapa K., Sampattavanich S., Akarasereenont P., Panich U. (2017). Activation of Nrf2 Reduces UVA-Mediated MMP-1 Upregulation via MAPK/AP-1 Signaling Cascades: The Photoprotective Effects of Sulforaphane and Hispidulin. J. Pharmacol. Exp. Ther..

[45] Wang J., Yuan M., Li Q., Shen C., Zhang X., Zhu C., Cen Q. (2025). Combined protection against UVB-induced photoaging by oleuropein, hydroxytyrosol, and verbascoside through modulation of inflammation, oxidative stress, and collagen homeostasis. Sci. Rep..

[46] Lin W.T., Chen Y.J., Kuo H.N., Kumar S., Abomughaid M.M., Senthil Kumar K.J. (2025). Ultraviolet B-induced oxidative damage in human skin keratinocytes is alleviated by Pinus morrisonicola leaf essential oil through activation of the Nrf2-dependent antioxidant defense system. Redox Rep..

[47] Liu Y., Wang R., Liu H., Tu Z. (2025). Dietary Phytochemicals Targeting NRF2 Against Skin Cellular Senescence: Mechanistic Insights and Potential for Functional Food Development. Biology.

[48] Krajka-Kuźniak V., Baer-Dubowska W. (2021). Modulation of Nrf2 and NF-κB Signaling Pathways by Naturally Occurring Compounds in Relation to Cancer Prevention and Therapy. Are Combinations Better Than Single Compounds?. Int. J. Mol. Sci..

[49] Ren Q., Qu L., Yuan Y., Wang F. (2024). Natural Modulators of Key Signaling Pathways in Skin Inflammaging. Clin. Cosmet. Investig. Dermatol..

[50] Sah A., Naseef P.P., Kuruniyan M.S., Jain G.K., Zakir F., Aggarwal G. (2022). A Comprehensive Study of Therapeutic Applications of Chamomile. Pharmaceuticals.

[51] Hu P., Li K., Peng X.X., Kan Y., Yao T.J., Wang Z.Y., Li Z., Liu H.Y., Cai D. (2023). Curcumin derived from medicinal homologous foods: Its main signals in immunoregulation of oxidative stress, inflammation, and apoptosis. Front. Immunol..

[52] Khan A.Q., Agha M.V., Sheikhan K.S.A.M., Younis S.M., Tamimi M.A., Alam M., Ahmad A., Uddin S., Buddenkotte J., Steinhoff M. (2022). Targeting deregulated oxidative stress in skin inflammatory diseases: An update on clinical importance. Biomed. Pharmacother..

[53] Piao M.J., Fernando P., Kang K.A., Fernando P., Herath H., Kim Y.R., Hyun J.W. (2024). Rosmarinic Acid Inhibits Ultraviolet B-Mediated Oxidative Damage via the AKT/ERK-NRF2-GSH Pathway In Vitro and In Vivo. Biomol. Ther..

[54] Stanescu C., Chiscop I., Mihalache D., Popa F., Tamas C., Stoleriu G. (2025). Skin Aging and Carotenoids: A Systematic Review of Their Multifaceted Protective Mechanisms. Nutrients.

[55] Flieger J., Raszewska-Famielec M., Radzikowska-Büchner E., Flieger W. (2024). Skin Protection by Carotenoid Pigments. Int. J. Mol. Sci..

[56] Ren Z.Q., Zheng S.Y., Sun Z., Luo Y., Wang Y.T., Yi P., Li Y.S., Huang C., Xiao W.F. (2025). Resveratrol: Molecular Mechanisms, Health Benefits, and Potential Adverse Effects. MedComm.

[57] Xia Y., Zhang H., Wu X., Xu Y., Tan Q. (2024). Resveratrol activates autophagy and protects from UVA-induced photoaging in human skin fibroblasts and the skin of male mice by regulating the AMPK pathway. Biogerontology.

[58] Xiu Y., Su Y., Gao L., Yuan H., Xu S., Liu Y., Qiu Y., Liu Z., Li Y. (2023). Corylin accelerated wound healing through SIRT1 and PI3K/AKT signaling: A candidate remedy for chronic non-healing wounds. Front. Pharmacol..

[59] Torno K.M.A., Tinio P.A.T., Lacson S.T.F. (2023). Efficacy of Oral Lycopene Supplementation for Photoprotection in Filipino Patients in a Tertiary Hospital in Makati: A Single-blind Randomized Controlled Trial. J. Philipp. Dermatol. Soc..

[60] Morilla M.J., Ghosal K., Romero E.L. (2023). More Than Pigments: The Potential of Astaxanthin and Bacterioruberin-Based Nanomedicines. Pharmaceutics.

[61] Sun J., Jiang Y., Fu J., He L., Guo X., Ye H., Yin C., Li H., Jiang H. (2024). Beneficial Effects of Epigallocatechin Gallate in Preventing Skin Photoaging: A Review. Molecules.

[62] Letsiou S., Koldiri E., Beloukas A., Rallis E., Kefala V. (2024). Deciphering the Effects of Different Types of Sunlight Radiation on Skin Function: A Review. Cosmetics.

[63] Budzianowska A., Banaś K., Budzianowski J., Kikowska M. (2025). Antioxidants to Defend Healthy and Youthful Skin—Current Trends and Future Directions in Cosmetology. Appl. Sci..

[64] Rudrapal M., Khairnar S.J., Khan J., Dukhyil A.B., Ansari M.A., Alomary M.N., Alshabrmi F.M., Palai S., Deb P.K., Devi R. (2022). Dietary Polyphenols and Their Role in Oxidative Stress-Induced Human Diseases: Insights Into Protective Effects, Antioxidant Potentials and Mechanism(s) of Action. Front. Pharmacol..

[65] Jomova K., Alomar S.Y., Alwasel S.H., Nepovimova E., Kuca K., Valko M. (2024). Several lines of antioxidant defense against oxidative stress: Antioxidant enzymes, nanomaterials with multiple enzyme-mimicking activities, and low-molecular-weight antioxidants. Arch. Toxicol..

[66] Tufail T., Bader Ul Ain H., Noreen S., Ikram A., Arshad M.T., Abdullahi M.A. (2024). Nutritional Benefits of Lycopene and Beta-Carotene: A Comprehensive Overview. Food Sci. Nutr..

[67] Li X., Matsumoto T., Takuwa M., Saeed Ebrahim Shaiku Ali M., Hirabashi T., Kondo H., Fujino H. (2020). Protective Effects of Astaxanthin Supplementation against Ultraviolet-Induced Photoaging in Hairless Mice. Biomedicines.

[68] Lima S.G.M., Freire M., Oliveira V.D.S., Solisio C., Converti A., De Lima Á.A.N. (2021). Astaxanthin Delivery Systems for Skin Application: A Review. Mar. Drugs.

[69] Ferrara F., Bondi A., Pula W., Contado C., Baldisserotto A., Manfredini S., Boldrini P., Sguizzato M., Montesi L., Benedusi M. (2024). Ethosomes for Curcumin and Piperine Cutaneous Delivery to Prevent Environmental-Stressor-Induced Skin Damage. Antioxidants.

[70] Michalak M. (2022). Plant-Derived Antioxidants: Significance in Skin Health and the Ageing Process. Int. J. Mol. Sci..

[71] Miao L., Daozhou L., Ying C., Qibing M., Siyuan Z. (2021). A resveratrol-loaded nanostructured lipid carrier hydrogel to enhance the anti-UV irradiation and anti-oxidant efficacy. Colloids Surf. B Biointerfaces.

[72] Mikled P., Chavasiri W., Khongkow M. (2024). Development of dihydrooxyresveratrol-loaded nanostructured lipid carriers for safe and effective treatment of hyperpigmentation. Sci. Rep..

[73] Mancuso A., Tarsitano M., Cavaliere R., Fresta M., Cristiano M.C., Paolino D. (2023). Gelled Liquid Crystal Nanocarriers for Improved Antioxidant Activity of Resveratrol. Gels.

[74] Hao B., Yang Z., Liu H., Liu Y., Wang S. (2024). Advances in flavonoid research: Sources, biological activities, and developmental prospectives. Curr. Issues Mol. Biol..

[75] Chen S., Wang X., Cheng Y., Gao H., Chen X. (2023). A Review of Classification, Biosynthesis, Biological Activities and Potential Applications of Flavonoids. Molecules.

[76] Zhou T., Zhang S., Zhou Y., Lai S., Chen Y., Geng Y., Wang J. (2021). Curcumin alleviates imiquimod-induced psoriasis in progranulin-knockout mice. Eur. J. Pharmacol..

[77] Zhuang W.B., Li Y.H., Shu X.C., Pu Y.T., Wang X.J., Wang T., Wang Z. (2023). The Classification, Molecular Structure and Biological Biosynthesis of Flavonoids, and Their Roles in Biotic and Abiotic Stresses. Molecules.

[78] Lai X., Li X., Chen J., Liu X., Pan P., Zhou Y., Zhao G. (2025). Advances in flavonoid glycosylation: Chemical and biological basis, mechanisms, physicochemical properties, and applications in the food industry. Trends Food Sci. Technol..

[79] Monmai C., Kim J.S., Chin J.H., Lee S., Baek S.H. (2023). Inhibitory Effects of Polyphenol- and Flavonoid-Enriched Rice Seed Extract on Melanogenesis in Melan-a Cells via MAPK Signaling-Mediated MITF Downregulation. Int. J. Mol. Sci..

[80] Velho P., Rebelo C.S., Macedo E.A. (2023). Extraction of Gallic Acid and Ferulic Acid for Application in Hair Supplements. Molecules.

[81] Alrumaihi F., Almatroodi S.A., Alharbi H.O.A., Alwanian W.M., Alharbi F.A., Almatroudi A., Rahmani A.H. (2024). Pharmacological Potential of Kaempferol, a Flavonoid in the Management of Pathogenesis via Modulation of Inflammation and Other Biological Activities. Molecules.

[82] Yoon J.H., Kim M.Y., Cho J.Y. (2023). Apigenin: A Therapeutic Agent for Treatment of Skin Inflammatory Diseases and Cancer. Int. J. Mol. Sci..

[83] Wiciński M., Erdmann J., Nowacka A., Kuźmiński O., Michalak K., Janowski K., Ohla J., Biernaciak A., Szambelan M., Zabrzyński J. (2023). Natural Phytochemicals as SIRT Activators—Focus on Potential Biochemical Mechanisms. Nutrients.

[84] Sharafan M., Dziki A., Malinowska M.A., Sikora E., Szopa A. (2025). Targeted Delivery Strategies for Hydrophilic Phytochemicals. Appl. Sci..

[85] Bialas I., Zelent-Kraciuk S., Jurowski K. (2023). The Skin Sensitisation of Cosmetic Ingredients: Review of Actual Regulatory Status. Toxics.

[86] Irizar A., Boislève F., Gautier F., Nash J.F., Pfuhler S., Ritacco G., Vey M., Wolf N., Cadby P.A. (2025). Phototoxicity and skin damage: A review of adverse effects of some furocoumarins found in natural extracts. Food Chem. Toxicol..

[87] Sindle A., Martin K. (2021). Art of Prevention: Essential Oils-Natural Products Not Necessarily Safe. Int. J. Women’s Dermatol..

[88] Kicińska A., Kowalczyk M. (2025). Health risks from heavy metals in cosmetic products available in the online consumer market. Sci. Rep..

[89] Khan S., Alam S., Siddiqui N., Azhar M., Rehman S. (2025). Heavy metals, aflatoxins, pesticide residues and microbial load determination in designed polyherbal formulation used against uterine fibroid. Discov. Chem..

[90] Kovač M., Bulaić M., Jakovljević J., Nevistić A., Rot T., Kovač T., Dodlek Šarkanj I., Šarkanj B. (2021). Mycotoxins, Pesticide Residues, and Heavy Metals Analysis of Croatian Cereals. Microorganisms.

[91] Caloni F., De Angelis I., Hartung T. (2022). Replacement of animal testing by integrated approaches to testing and assessment (IATA): A call for in vivitrosi. Arch. Toxicol..

[92] Gądarowska D., Kalka J., Daniel-Wójcik A., Mrzyk I. (2022). Alternative Methods for Skin-Sensitization Assessment. Toxics.

[93] Bernauer U., Bodin L., Chaudhry Q., Coenraads P., Dusinska M., Ezendam J., Gaffet E., Galli C., Panteri E., Rogiers V. (2023). SCCS Notes of Guidance for the Testing of Cosmetic Ingredients and Their Safety Evaluation-12th Revision-Final Opinion–SCCS/1647/22-Corrigendum 2.

[94] Organization for Economic Co-Operation and Development (2022). Test No. 442D: In Vitro skin Sensitisation: ARE-Nrf2 Luciferase Test Method, OECD GUIDELINES for the Testing of Chemicals, Section 4.

[95] Martins R., Barbosa A., Advinha B., Sales H., Pontes R., Nunes J. (2023). Green Extraction Techniques of Bioactive Compounds: A State-of-the-Art Review. Processes.

[96] Palos-Hernández A., González-Paramás A.M., Santos-Buelga C. (2025). Latest Advances in Green Extraction of Polyphenols from Plants, Foods and Food By-Products. Molecules.

[97] Lee J.H., Park J., Shin D.W. (2022). The Molecular Mechanism of Polyphenols with Anti-Aging Activity in Aged Human Dermal Fibroblasts. Molecules.

[98] Nan L., Guo P., Hui W., Xia F., Yi C. (2025). Recent advances in dermal fibroblast senescence and skin aging: Unraveling mechanisms and pioneering therapeutic strategies. Front. Pharmacol..

[99] Ferreira M., Matos A., Couras A., Marto J., Ribeiro H. (2022). Overview of Cosmetic Regulatory Frameworks around the World. Cosmetics.

[100] Manful M.E., Ahmed L., Barry-Ryan C. (2024). Cosmetic formulations from natural sources: Safety considerations and legislative frameworks in the European Union. Cosmetics.

[101] Bouwstra J.A., Nădăban A., Bras W., McCabe C., Bunge A., Gooris G.S. (2023). The skin barrier: An extraordinary interface with an exceptional lipid organization. Prog. Lipid Res..

[102] Erdoğar N., Gür B., Örgül D. (2026). Recent developments of novel nanotechnology-based drug delivery systems for dermal and transdermal applications. Eur. J. Pharm. Sci..

[103] Raina N., Rani R., Thakur V.K., Gupta M. (2023). New Insights in Topical Drug Delivery for Skin Disorders: From a Nanotechnological Perspective. ACS Omega.

[104] Jonsdottir F., Snorradottir B.S., Gunnarsson S., Georgsdottir E., Sigurdsson S. (2022). Transdermal Drug Delivery: Determining Permeation Parameters Using Tape Stripping and Numerical Modeling. Pharmaceutics.

[105] Pulsoni I., Lubda M., Aiello M., Fedi A., Marzagalli M., von Hagen J., Scaglione S. (2022). Comparison Between Franz Diffusion Cell and a novel Micro-physiological System for In Vitro Penetration Assay Using Different Skin Models. SLAS Technol..

[106] Iliopoulos F., Caspers P.J., Puppels G.J., Lane M.E. (2020). Franz Cell Diffusion Testing and Quantitative Confocal Raman Spectroscopy: In Vitro-In Vivo Correlation. Pharmaceutics.

[107] Safta D.A., Bogdan C., Moldovan M.-L. (2024). SLNs and NLCs for Skin Applications: Enhancing the Bioavailability of Natural Bioactives. Pharmaceutics.

[108] Mancuso A., Cristiano M.C., Pandolfo R., Greco M., Fresta M., Paolino D. (2021). Improvement of Ferulic Acid Antioxidant Activity by Multiple Emulsions: In Vitro and In Vivo Evaluation. Nanomaterials.

[109] Pereira A., Ramalho M.J., Silva R., Silva V., Marques-Oliveira R., Silva A.C., Pereira M.C., Loureiro J.A. (2022). Vine Cane Compounds to Prevent Skin Cells Aging through Solid Lipid Nanoparticles. Pharmaceutics.

[110] Li D., Martini N., Wu Z., Chen S., Falconer J.R., Locke M., Zhang Z., Wen J. (2022). Niosomal Nanocarriers for Enhanced Dermal Delivery of Epigallocatechin Gallate for Protection against Oxidative Stress of the Skin. Pharmaceutics.

[111] Upare M.M., Thorawade K.M., Mishra A.P., Nigam M., Waranuch N. (2025). Lipid-based nanocarriers in topical applications for skin infections. Curr. Opin. Pharmacol..

[112] Friedman N., Merims S., Elia J., Benny O. (2022). Ex-vivo Skin Permeability Tests of Nanoparticles for Microscopy Imaging. Bio-Protocol.

[113] Imran M., Iqubal M.K., Imtiyaz K., Saleem S., Mittal S., Rizvi M.M.A., Ali J., Baboota S. (2020). Topical nanostructured lipid carrier gel of quercetin and resveratrol: Formulation, optimization, in vitro and ex vivo study for the treatment of skin cancer. Int. J. Pharm..

[114] Lúcio M., Giannino N., Barreira S., Catita J., Gonçalves H., Ribeiro A., Fernandes E., Carvalho I., Pinho H., Cerqueira F. (2023). Nanostructured Lipid Carriers Enriched Hydrogels for Skin Topical Administration of Quercetin and Omega-3 Fatty Acid. Pharmaceutics.

[115] de Barros D.P.C., Santos R., Reed P., Fonseca L.P., Oliva A. (2022). Design of Quercetin-Loaded Natural Oil-Based Nanostructured Lipid Carriers for the Treatment of Bacterial Skin Infections. Molecules.

[116] Arora D., Nanda S. (2019). Quality by design driven development of resveratrol loaded ethosomal hydrogel for improved dermatological benefits via enhanced skin permeation and retention. Int. J. Pharm..

[117] Richard C., Cassel S., Blanzat M. (2020). Vesicular systems for dermal and transdermal drug delivery. RSC Adv..

[118] Seenivasan R., Halagali P., Nayak D., Tippavajhala V.K. (2025). Transethosomes: A Comprehensive Review of Ultra-Deformable Vesicular Systems for Enhanced Transdermal Drug Delivery. AAPS PharmSciTech.

[119] Malviya N., A P., Alexander A. (2023). Comparative study on ethosomes and transferosomes for enhancing skin permeability of sinapic acid. J. Mol. Liq..

[120] Lapmanee S., Bunwatcharaphansakun P., Phongsupa W., Namdee K., Suttisintong K., Asawapirom U., Ruktanonchai U., Wongchitrat P., Bhubhanil S., Maitarad P. (2025). Transfersomal delivery of Centella asiatica promotes efficient excision wound healing in rats. Drug Deliv..

[121] Ciriolo L., d’Avanzo N., Mancuso A., Cristiano M.C., Barone A., Mare R., Tolomeo A.M., Comaniciu A.I., Nitulescu G., Olaru O.T. (2025). Microfluidic Design of Ultradeformable Liposomes for Advanced Skin Delivery of Stellaria media Phytocomplex. Pharmaceutics.

[122] Kim M.K., Choi Y.C., Cho S.H., Choi J.S., Cho Y.W. (2021). The Antioxidant Effect of Small Extracellular Vesicles Derived from Aloe vera Peels for Wound Healing. Tissue Eng. Regen. Med..

[123] Di Raimo R., Mizzoni D., Aloi A., Pietrangelo G., Dolo V., Poppa G., Fais S., Logozzi M. (2024). Antioxidant Effect of a Plant-Derived Extracellular Vesicles’ Mix on Human Skin Fibroblasts: Induction of a Reparative Process. Antioxidants.

[124] Jianda X., Zimo Y., Yuhan D., Zhongyu X., Kewei Z., Xiaolan C. (2025). Plant-derived extracellular vesicles in diabetic wound healing: Mechanisms, therapeutic implications and future perspectives. J. Mater. Sci. Mater. Med..

[125] Souto E.B., Fangueiro J.F., Fernandes A.R., Cano A., Sanchez-Lopez E., Garcia M.L., Severino P., Paganelli M.O., Chaud M.V., Silva A.M. (2022). Physicochemical and biopharmaceutical aspects influencing skin permeation and role of SLN and NLC for skin drug delivery. Heliyon.

[126] Bernauer U., Bodin L., Chaudhry Q., Coenraads P.J., Dusinska M., Gaffet E., Panteri E., Rogiers V., Rousselle C., Stepnik M. (2021). The SCCS scientific advice on the safety of nanomaterials in cosmetics. Regul. Toxicol. Pharmacol..

[127] Coimbra S.C., Sousa-Oliveira I., Ferreira-Faria I., Peixoto D., Pereira-Silva M., Mathur A., Pawar K.D., Raza F., Mazzola P.G., Mascarenhas-Melo F. (2022). Safety Assessment of Nanomaterials in Cosmetics: Focus on Dermal and Hair Dyes Products. Cosmetics.

[128] Han J., Kim S., Lee S.H., Kim J.S., Chang Y.J., Jeong T.C., Kang M.J., Kim T.S., Yoon H.S., Lee G.Y. (2020). Me-too validation study for in vitro skin irritation test with a reconstructed human epidermis model, KeraSkin™ for OECD test guideline 439. Regul. Toxicol. Pharmacol. RTP.

[129] Cronin M.T.D., Enoch S.J., Madden J.C., Rathman J.F., Richarz A.-N., Yang C. (2022). A review of in silico toxicology approaches to support the safety assessment of cosmetics-related materials. Comput. Toxicol..

[130] Nie Y., Li Y. (2025). Curcumin: A potential anti-photoaging agent. Front. Pharmacol..

[131] Draelos Z.D., Gunt H., Zeichner J., Levy S. (2020). Clinical Evaluation of a Nature-Based Bakuchiol Anti-Aging Moisturizer for Sensitive Skin. J. Drugs Dermatol. JDD.

[132] Alberts A., Lungescu I.A., Niculescu A.-G., Grumezescu A.M. (2025). Natural Products for Improving Soft Tissue Healing: Mechanisms, Innovations, and Clinical Potential. Pharmaceutics.

[133] Naharro-Rodriguez J., Bacci S., Hernandez-Bule M.L., Perez-Gonzalez A., Fernandez-Guarino M. (2025). Decoding Skin Aging: A Review of Mechanisms, Markers, and Modern Therapies. Cosmetics.

[134] Nobile V., Schiano I., Peral A., Giardina S., Spartà E., Caturla N. (2021). Antioxidant and reduced skin-ageing effects of a polyphenol-enriched dietary supplement in response to air pollution: A randomized, double-blind, placebo-controlled study. Food Nutr. Res..

[135] Farhan M. (2024). The Promising Role of Polyphenols in Skin Disorders. Molecules.

[136] Ikeda Y., Nasu M., Bruxer J.Y., Díaz-Puertas R., Martínez-Godfrey J., Bulbiankova D., Herranz-López M., Micol V., Álvarez-Martínez F.J. (2025). Photoprotective, Antioxidant and Anti-Inflammatory Effects of Aged Punica granatum Extract: In Vitro and In Vivo Insights. Food Sci. Nutr..

[137] Sykuła A., Janiak-Włodarczyk I., Kapusta I.T. (2025). Formulation and Evaluation of the Antioxidant Activity of an Emulsion Containing a Commercial Green Tea Extract. Molecules.

[138] Ye H., Sun J., He L., Ai C., Jin W., Abd El-Aty A.M. (2025). Beneficial effects of proanthocyanidins on skin aging: A review. Front. Nutr..

[139] Chen P.Y., Tu Y.X., Wu C.T., Jong T.T., Chang C.M.J. (2004). Continuous Hot Pressurized Solvent Extraction of 1,1-Diphenyl-2-Picrylhydrazyl Free Radical Scavenging Compounds from Taiwan Yams (Dioscorea Alata). J. Agric. Food Chem..

[140] Liu J., Xu X., Jian M., Guo Y., Zhai L., Sun G., Sun L., Jiang R. (2025). Glycyrrhiza glabra extract as a skin-whitening Agent: Identification of active components and CRTC1/MITF pathway-inhibition mechanism. J. Ethnopharmacol..

[141] Namiecińska E., Jaszczak J., Hikisz P., Daśko M., Woźniczka M., Budzisz E. (2025). Evaluation of Tyrosinase Inhibitory Activity of Carbathioamidopyrazoles and Their Potential Application in Cosmetic Products and Melanoma Treatment. Int. J. Mol. Sci..

[142] Yang H.L., Lin C.P., Vudhya Gowrisankar Y., Huang P.J., Chang W.L., Shrestha S., Hseu Y.C. (2021). The anti-melanogenic effects of ellagic acid through induction of autophagy in melanocytes and suppression of UVA-activated α-MSH pathways via Nrf2 activation in keratinocytes. Biochem. Pharmacol..

[143] Amini N., Osterlund C., Curpen J., Lafon-Kolb V., Richard T., Visdal-Johnsen L. (2025). Phytoestrogens as Natural Anti-Aging Solutions for Enhanced Collagen Synthesis in Skin. J. Cosmet. Dermatol..

[144] Câmara J.S., Locatelli M., Pereira J.A.M., Oliveira H., Arlorio M., Fernandes I., Perestrelo R., Freitas V., Bordiga M. (2022). Behind the Scenes of Anthocyanins—From the Health Benefits to Potential Applications in Food, Pharmaceutical and Cosmetic Fields. Nutrients.

[145] Jing R., Fu M., Huang Y., Zhang K., Ye J., Gong F., Jihea Ali Naji Nasser A.B., Xu X., Xiao J., Yu G. (2024). Oat β-glucan repairs the epidermal barrier by upregulating the levels of epidermal differentiation, cell-cell junctions and lipids via Dectin-1. Br. J. Pharmacol..

[146] Matei C.E., Visan A.I., Cristescu R. (2025). Aloe Vera Polysaccharides as Therapeutic Agents: Benefits Versus Side Effects in Biomedical Applications. Polysaccharides.

[147] Shafaie S., Andalib S., Shafaei H., Montaseri A., Tavakolizadeh M. (2020). Differential Biological Behavior of Fibroblasts and Endothelial Cells under Aloe vera Gel Culturing. Int. J. Mol. Cell. Med..

[148] Arribas-López E., Zand N., Ojo O., Snowden M.J., Kochhar T. (2022). A Systematic Review of the Effect of *Centella asiatica* on Wound Healing. Int. J. Environ. Res. Public Health.

[149] Sun B., Wu L., Wu Y., Zhang C., Qin L., Hayashi M., Kudo M., Gao M., Liu T. (2020). Therapeutic Potential of *Centella asiatica* and Its Triterpenes: A Review. Front. Pharmacol..

[150] de Macedo L.M., Santos É.M.d., Militão L., Tundisi L.L., Ataide J.A., Souto E.B., Mazzola P.G. (2020). Rosemary (*Rosmarinus officinalis* L., syn *Salvia rosmarinus* Spenn.) and Its Topical Applications: A Review. Plants.

[151] El-Sakhawy M., Salama A., Tohamy H.S. (2023). Applications of propolis-based materials in wound healing. Arch. Dermatol. Res..

[152] Loya-Hernández L.P., Arzate-Quintana C., Castillo-González A.R., Camarillo-Cisneros J., Romo-Sáenz C.I., Favila-Pérez M.A., Quiñonez-Flores C.M. (2025). Propolis-Functionalized Biomaterials for Wound Healing: A Systematic Review with Emphasis on Polysaccharide-Based Platforms. Polysaccharides.

[153] Gendrisch F., Esser P.R., Schempp C.M., Wölfle U. (2021). Luteolin as a modulator of skin aging and inflammation. Biofactors.

[154] Zhang B., Wang J., Zhao G., Lin M., Lang Y., Zhang D., Feng D., Tu C. (2020). Apigenin protects human melanocytes against oxidative damage by activation of the Nrf2 pathway. Cell Stress Chaperones.

[155] Barak T.H., Eryilmaz M., Karaca B., Servi H., Kara Ertekin S., Dinc M., Ustuner H. (2025). Antimicrobial, Anti-Biofilm, Anti-Quorum Sensing and Cytotoxic Activities of *Thymbra spicata* L. subsp. *spicata* Essential Oils. Antibiotics.

[156] Bhatia E., Sharma S., Jadhav K., Banerjee R. (2021). Combinatorial liposomes of berberine and curcumin inhibit biofilm formation and intracellular methicillin resistant *Staphylococcus aureus* infections and associated inflammation. J. Mater. Chem. B.

[157] Bowbe K.H., Salah K.B.H., Moumni S., Ashkan M.F., Merghni A. (2023). Anti-Staphylococcal Activities of *Rosmarinus officinalis* and *Myrtus communis* Essential Oils through ROS-Mediated Oxidative Stress. Antibiotics.

[158] Nurzyńska-Wierdak R., Pietrasik D., Walasek-Janusz M. (2023). Essential Oils in the Treatment of Various Types of Acne—A Review. Plants.

[159] Wang J., Zhang L., Fu L., Pang Z. (2025). Kaempferol Mitigates *Pseudomonas aeruginosa*-Induced Acute Lung Inflammation Through Suppressing GSK3β/JNK/c-Jun Signaling Pathway and NF-κB Activation. Pharmaceuticals.

[160] Kim H.J., Kim Y.H. (2024). Exploring Acne Treatments: From Pathophysiological Mechanisms to Emerging Therapies. Int. J. Mol. Sci..

[161] Leti M., Garidou L., Cuisiat S.V., Stennevin A., Doat G., Mias C. (2025). Plant Extracts in Acne Management: A Narrative Review. Dermatology.

[162] Salazar J., Ortega Á., Pérez J.L., Garrido B., Santeliz R., Galbán N., Díaz M.P., Cano R., Cano G., Contreras-Velasquez J.C. (2025). Role of polyphenols in dermatological diseases: Exploring pharmacotherapeutic mechanisms and clinical implications. Pharmaceuticals.

[163] Zheng X.Q., Zhang X.H., Gao H.Q., Huang L.Y., Ye J.J., Ye J.H., Lu J.L., Lu J.L., Ma S.C., Liang Y.R. (2024). Green Tea Catechins and Skin Health. Antibiotics.

[164] Sun C., Na Y., Wang Z., Zhu T., Liu X. (2024). Phytochemicals, promising strategies combating Cutibacterium acnes. Front. Pharmacol..

[165] Kairey L., Agnew T., Bowles E.J., Barkla B.J., Wardle J., Lauche R. (2023). Efficacy and safety of *Melaleuca alternifolia* (tea tree) oil for human health—A systematic review of randomized controlled trials. Front. Pharmacol..

[166] Koch W., Zagórska J., Michalak-Tomczyk M., Karav S., Wawruszak A. (2024). Plant Phenolics in the Prevention and Therapy of Acne: A Comprehensive Review. Molecules.

[167] Liu J., Guan Y., Yang L., Fang H., Sun H., Sun Y., Yan G., Kong L., Wang X. (2025). Ferulic Acid as an Anti-Inflammatory Agent: Insights into Molecular Mechanisms, Pharmacokinetics and Applications. Pharmaceuticals.

[168] Zheng N., Xie Y., Zhou M., Liu Y., Xu H., Zeng R., Wan C., Li M. (2024). Utilizing the photodynamic properties of curcumin to disrupt biofilms in *Cutibacterium acnes*: A promising approach for treating acne. Photodiagn. Photodyn. Ther..

[169] Wang X., Jia Y., He H. (2024). The Role of Linoleic Acid in Skin and Hair Health: A Review. Int. J. Mol. Sci..

[170] Kim H.-D., Choi H., Abekura F., Park J.-Y., Yang W.-S., Yang S.-H., Kim C.-H. (2023). Naturally-Occurring Tyrosinase Inhibitors Classified by Enzyme Kinetics and Copper Chelation. Int. J. Mol. Sci..

[171] Fowler J.F., Ma L., Bergman J., Horowitz P., Lavender T., Eichenfield L.F., Draelos Z., Danby S.G., Cork M.J. (2025). Is colloidal oat an effective emollient ingredient for the prevention and treatment of atopic dermatitis in infants?. J. Dermatol. Treat..

[172] Xu J., Hu H., Qian X., Zhang D., Chen G., Zhang F., Huang X., Ma S., Chen B., Zhou Q. (2024). Therapeutic effects of chamomile volatile oil nanoemulsion/*Bletilla striata* polysaccharides gels on atopic dermatitis. Int. J. Biol. Macromol..

[173] Chelu M., Musuc A.M., Popa M., Calderon Moreno J. (2023). Aloe vera-Based Hydrogels for Wound Healing: Properties and Therapeutic Effects. Gels.

[174] Bîrsan M., Gore E., Scripcariu Ș.-I., Vlad R.-A., Antonoaea P., Pintea C., Pintea A., Cotoi C.-T., Focșa A.-V., Ciurba A. (2025). Multi-Active Cosmeceutical Formulations: Stability, Sensory Performance, and Skin Tolerability. Cosmetics.

[175] Rischard F., Gore E., Flourat A., Savary G. (2025). The challenges faced by multifunctional ingredients: A critical review from sourcing to cosmetic applications. Adv. Colloid Interface Sci..

[176] Chong J.R., De Lucia C., Tovar-Rios D.A., Castellanos-Perilla N., Collins C., Kvernberg S.M., Ballard C., Siow R.C., Aarsland D. (2024). A Randomised, Double-Blind, Placebo-Controlled, Cross-Over Clinical Trial to Evaluate the Biological Effects and Safety of a Polyphenol Supplement on Healthy Ageing. Antioxidants.

[177] Christman L., De Benedetto A., Johnson E., Khoo C., Gu L. (2024). Polyphenol-Rich Cranberry Beverage Positively Affected Skin Health, Skin Lipids, Skin Microbiome, Inflammation, and Oxidative Stress in Women in a Randomized Controlled Trial. Nutrients.

[178] Cheng F., Feng J., Cao Z., Duan Q., Li H. (2025). Efficacy and Safety of Topical Application of Plant-Based Products on Skin Aging in Healthy Individuals: A Systematic Review and Meta-Analysis of Randomized Controlled Trials. J. Cosmet. Dermatol..

[179] Nobile V., Schiano I., Germani L., Cestone E., Navarro P., Jones J., Caturla N. (2023). Skin Anti-Aging Efficacy of a Four-Botanical Blend Dietary Ingredient: A Randomized, Double Blind, Clinical Study. Cosmetics.

[180] Crespi O., Rosset F., Pala V., Sarda C., Accorinti M., Quaglino P., Ribero S. (2025). Cosmeceuticals for Anti-Aging: Mechanisms, Clinical Evidence, and Regulatory Insights—A Comprehensive Review. Cosmetics.

[181] Proksch E., Berardesca E., Misery L., Engblom J., Bouwstra J. (2020). Dry skin management: Practical approach in light of latest research on skin structure and function. J. Dermatol. Treat..

[182] Pezantes-Orellana C., German Bermúdez F., Montalvo J., Packer T., Orellana-Manzano A. (2025). Evaluating efficacy, safety, and innovation in skin care applications of essential oils: A systematic review. Front. Med..

[183] Potvin D., D’Angelo P., Bennett S., Jankicevic J., Bissonnette R. (2024). Adaptive designs in dermatology clinical trials: Current status and future perspectives. J. Eur. Acad. Dermatol. Venereol..

[184] Sivamani R.K., Ablon G., Nong Y., Maloh J., Hazan A., Raymond I. (2024). A Prospective, Multi-Center Study to Evaluate the Safety and Efficacy of a Vegan Nutraceutical to Improve Hair Growth and Quality in Females Following a Plant-Based Diet. J. Drugs Dermatol. JDD.

[185] Aguiar J.B., Martins A.M., Almeida C., Ribeiro H.M., Marto J. (2022). Water sustainability: A waterless life cycle for cosmetic products. Sustain. Prod. Consum..

[186] Val S., Lambán M.P. (2025). Enhancing Sustainability with LCA: A Comparative Analysis of Design and Manufacturing Processes. Processes.

[187] Silletta A., Mancuso A., d’Avanzo N., Cristiano M.C., Paolino D. (2024). Antimicrobial Compounds from Food Waste in Cosmetics. Cosmetics.

[188] d’Avanzo N., Mancuso A., Mare R., Silletta A., Maurotti S., Parisi O.I., Cristiano M.C., PaolinoD (2024). Olive Leaves and Citrus Peels: From Waste to Potential Resource for Cosmetic Products. Cosmetics.

[189] Bubulac L., Bogdan-Andreescu C.F., Voica D.V., Cristea B.M., Chiș M.S., Slăvescu D.A. (2025). From Olive Oil to Pomace: Sustainable Valorization Pathways Linking Food Processing and Human Health. Appl. Sci..

[190] Nunes A., Marto J., Gonçalves L., Martins A.M., Fraga C., Ribeiro H.M. (2021). Potential therapeutic of olive oil industry by-products in skin health: A review. Int. J. Food Sci. Technol..

[191] Selim S., Albqmi M., Al-Sanea M.M., Alnusaire T.S., Almuhayawi M.S., AbdElgawad H., Al Jaouni S.K., Elkelish A., Hussein S., Warrad M. (2022). Valorizing the usage of olive leaves, bioactive compounds, biological activities, and food applications: A comprehensive review. Front. Nutr..

[192] Kontaxi N.-I., Panoutsopoulou E., Ofrydopolou A., Tsoupras A. (2024). Anti-Inflammatory Benefits of Grape Pomace and Tomato Bioactives as Ingredients in Sun Oils against UV Radiation for Skin Protection. Appl. Sci..

[193] Szabo K., Varvara R.-A., Ciont C., Macri A.M., Vodnar D.C. (2025). An updated overview on the revalorization of bioactive compounds derived from tomato production and processing by-products. J. Clean. Prod..

[194] Bhutani M., Gaur S.S., Shams R., Dash K.K., Shaikh A.M., Béla K. (2025). Valorization of grape by-products: Insights into sustainable industrial and nutraceutical applications. Future Foods.

[195] Machado T.O.X., Portugal I., Kodel H.d.A.C., Droppa-Almeida D., Dos Santos Lima M., Fathi F., Oliveira M.B.P.P., de Albuquerque-Júnior R.L.C., Dariva C., Souto E.B. (2024). Therapeutic potential of grape pomace extracts: A review of scientific evidence. Food Biosci..

[196] Beltran M., Tjahjono B., Bogush A., Julião J., Teixeira E.L.S. (2021). Food Plastic Packaging Transition towards Circular Bioeconomy: A Systematic Review of Literature. Sustainability.

[197] Vrabič-Brodnjak U., Jestratijević I. (2024). The future of baby cosmetics packaging and sustainable development: A look at sustainable materials and packaging innovations—A systematic review. Sustain. Dev..

[198] Asim Z., Shamsi I.R.A., Wahaj M., Raza A., Abul Hasan S., Siddiqui S.A., Aladresi A., Sorooshian S., Seng Teck T. (2022). Significance of Sustainable Packaging: A Case-Study from a Supply Chain Perspective. Appl. Syst. Innov..

[199] Chebanova I. (2025). Interdisciplinary Study of the Impact of Environmental Trends and Sustainability Requirements on the Strategic Evolution of the Cosmetics Industry—From Packaging Ecodesign and Green Chemistry to the Transformation of Supply Chains, Marketing Practices, and Regulatory Compliance—With an Econometric Assessment of Market Dynamics and Consumer Preferences. Univers. Libr. Innov. Res. Stud..

[200] Martins A.M., Silva A.T., Marto J.M. (2025). Advancing Cosmetic Sustainability: Upcycling for a Circular Product Life Cycle. Sustainability.

[201] Lin Y., Wang Y., Li Y. (2025). Exploring alternative solvents to n-hexane for green extraction of lipid from camellia oil cakes. Food Chem. X.

[202] Bozza A., Campi C., Garelli S., Ugazio E., Battaglia L. (2022). Current regulatory and market frameworks in green cosmetics: The role of certification. Sustain. Chem. Pharm..

[203] Liu X., Yahya Dawod A. (2025). When Technology Signals Trust: Blockchain vs. Traditional Cues in Cross-Border Cosmetic E-Commerce. Information.

[204] Castro L.E.N., Sganzerla W.G., Silva A.P.G., John O.D., Barroso T.L.C.T., Rostagno M.A., Forster-Carneiro T. (2025). Sustainable extraction methods for the recovery of polyphenolic compounds from grape pomace and its biological properties: A comprehensive review. Phytochem. Rev..

[205] Frontini A., Luvisi A., Negro C., Apollonio M., Accogli R., De Pascali M., De Bellis L. (2024). Polyphenols Extraction from Different Grape Pomaces Using Natural Deep Eutectic Solvents. Separations.

[206] Fernandes F., Delerue-Matos C., Grosso C. (2025). Unveiling the potential of agrifood by-products: A comprehensive review of phytochemicals, bioactivities and industrial applications. Waste Biomass Valorization.

[207] Saini R.K., Khan M.I., Kumar V., Shang X., Lee J.H., Ko E.Y. (2025). Bioactive Compounds of Agro-Industrial By-Products: Current Trends, Recovery, and Possible Utilization. Antioxidants.

[208] Dutta D., Sit N. (2024). A comprehensive review on types and properties of biopolymers as sustainable bio-based alternatives for packaging. Food Biomacromol..

[209] Omira A., Grira S., Mourad A.-H.I., Alkhedher M. (2025). The new generation of cosmetics packaging: A paradigm shift. Glob. Transit..

[210] Perera K.Y., Jaiswal A.K., Jaiswal S. (2023). Biopolymer-Based Sustainable Food Packaging Materials: Challenges, Solutions, and Applications. Foods.

[211] Ogorzałek M., Klimaszewska E., Małysa A., Czerwonka D., Tomasiuk R. (2024). Research on Waterless Cosmetics in the Form of Scrub Bars Based on Natural Exfoliants. Appl. Sci..

[212] Martins A.M., Marto J.M. (2023). A sustainable life cycle for cosmetics: From design and development to post-use phase. Sustain. Chem. Pharm..

[213] Kakran S., Rathore J.S., Sidhu A., Kumar A. (2024). Solar energy advances and CO_2_ emissions: A comparative review of leading nations’ path to sustainable future. J. Clean. Prod..

[214] Kabaja B., Wojnarowska M., Ćwiklicki M., Buffagni S.C., Varese E. (2023). Does environmental labelling still matter? Generation Z’s purchasing decisions. Sustainability.

[215] Ahmed W.A.H., MacCarthy B.L. (2023). Blockchain-enabled supply chain traceability—How wide? How deep?. Int. J. Prod. Econ..

[216] Kaestner L., Scope C., Neumann N., Woelfel C. (2023). Sustainable circular packaging design: A systematic literature review on strategies and applications in the cosmetics industry. Proc. Des. Soc..

[217] Mondello A., Salomone R., Mondello G. (2024). Exploring circular economy in the cosmetic industry: Insights from a literature review. Environ. Impact Assess. Rev..

[218] Rocca R., Acerbi F., Fumagalli L., Taisch M. (2023). Development of an LCA-based tool to assess the environmental sustainability level of cosmetics products. Int. J. Life Cycle Assess..

[219] Rudolf R., Majerič P., Pintarič Z.N., Horvat A., Krajnc D. (2024). Life Cycle Assessment (LCA) of the Impact on the Environment of a Cosmetic Cream with Gold Nanoparticles and Hydroxylated Fullerene Ingredients. Appl. Sci..

[220] U.S. Food and Drug Administration (FDA) (2018). Key Legal Concepts for Cosmetics Industry: Interstate Commerce, Adulterated, and Misbranded.

[221] Bindusahithi L., Keerthika V., Lakshmi V.N., Rajeswari A. (2025). A Comprehensive Study on Quasi-Drugs Regulations in Japan and South Korea. Int. J. Res. Publ. Rev..

[222] Su Z., Luo F.-y., Pei X.-r., Zhang F.-l., Xing S.-x., Wang G.-l. (2020). Final Publication of the “Regulations on the Supervision and Administration of Cosmetics” and New Prospectives of Cosmetic Science in China. Cosmetics.

[223] Pistollato F., Madia F., Corvi R., Munn S., Grignard E., Paini A., Worth A., Bal-Price A., Prieto P., Casati S. (2021). Current EU regulatory requirements for the assessment of chemicals and cosmetic products: Challenges and opportunities for introducing new approach methodologies. Arch. Toxicol..

[224] da Silva J.D., Silva F.A.M., Rodrigues C.F. (2025). Microbial Contamination in Cosmetic Products. Cosmetics.

[225] Kim H.W., Seok Y.S., Cho T.J., Rhee M.S. (2020). Risk factors influencing contamination of customized cosmetics made on-the-spot: Evidence from the national pilot project for public health. Sci. Rep..

[226] Kirkbride L., Humphries L., Kozielska P., Curtis H. (2021). Designing a Suitable Stability Protocol in the Face of a Changing Retail Landscape. Cosmetics.

[227] Serra M., Botelho C., Almeida H., Casas A., Teixeira J.A., Barros A.N. (2025). Stable and Functional Cosmetic Creams Enriched with Grape Stem Extract: A Sustainable Skincare Strategy. Antioxidants.

[228] Commission E. (2013). Commission Regulation (EU) No 655/2013 Laying Down Common Criteria for the Justification of Claims Used in Relation to Cosmetic Products.

[229] Intertek Group plc Understanding the EU Cosmetic Regulation and Attaining Compliance. https://www.intertek.com/resources/white-papers/2016/understanding-the-eu-cosmetics-regulation-white-paper/.

[230] Brescia S., Alexander-White C., Li H., Cayley A. (2023). Risk assessment in the 21st century: Where are we heading?. Toxicol. Res..

[231] Herzler M., Abedini J., Allen D.G., Germolec D., Gordon J., Ko H.S., Matheson J., Reinke E., Strickland J., Thierse H.J. (2024). Use of human predictive patch test (HPPT) data for the classification of skin sensitization hazard and potency. Arch. Toxicol..

[232] OECD (2017). Guidance Document on the Reporting of Defined Approaches and Individual Information Sources to be Used Within Integrated Approaches to Testing and Assessment (IATA).

[233] Basketter D.A., Gerberick G.F. (2022). Skin Sensitization Testing: The Ascendancy of Non-Animal Methods. Cosmetics.

[234] OECD (2025). Guideline No. 497: Defined Approaches on Skin Sensitisation.

[235] Lai M.B., Vergamini D., Brunori G. (2025). Food Supply Chain: A Framework for the Governance of Digital Traceability. Foods.

[236] Wittwehr C., Blomstedt P., Gosling J.P., Peltola T., Raffael B., Richarz A.-N., Sienkiewicz M., Whaley P., Worth A., Whelan M. (2020). Artificial Intelligence for chemical risk assessment. Comput. Toxicol..

[237] Rudroff T. (2024). Artificial Intelligence as a Replacement for Animal Experiments in Neurology: Potential, Progress, and Challenges. Neurol. Int..

[238] Sala S., Amadei A.M., Beylot A., Ardente F. (2021). The evolution of life cycle assessment in European policies over three decades. Int. J. Life Cycle Assess..

[239] Kaynak E., Piri I.S., Das O. (2025). Revisiting the Basics of Life Cycle Assessment and Lifecycle Thinking. Sustainability.

